# Prophylactic exercise-derived circulating exosomal miR-125a-5p promotes endogenous revascularization after hindlimb ischemia by targeting endothelin converting enzyme 1

**DOI:** 10.3389/fcvm.2022.881526

**Published:** 2022-07-22

**Authors:** Xueting Qiu, Jipeng Zhou, Yanying Xu, Longsheng Liao, Huijun Yang, Yuan Xiang, Zhengshi Zhou, Quan Sun, Minghong Chen, Jiaxiong Zhang, Wanzhou Wu, Lingping Zhu, Baiyang You, Lingfang He, Ying Luo, Zhenyu Li, Chuanchang Li, Yongping Bai

**Affiliations:** ^1^Department of Geriatric Medicine, Xiangya Hospital, Central South University, Changsha, China; ^2^National Clinical Research Center for Geriatric Disorders, Xiangya Hospital, Central South University, Changsha, China; ^3^Department of Cardiovascular Medicine, Xiangya Hospital, Central South University, Changsha, China; ^4^Department of Laboratory Animal, Xiangya School of Medicine, Central South University, Changsha, China

**Keywords:** exercise, miR-125a-5p, circulating, exosome, revascularization

## Abstract

**Background:**

Prophylactic exercise improves clinical outcomes in patients experiencing severe ischemic diseases. Previous studies have shown that exercise could alter the amount or content of circulating exosomes. However, little is known about the role of precursory exercise-derived circulating exosomes (Exe-Exo) in ischemic diseases. We therefore aimed to explore the function and mechanism of Exe-Exo in endogenous revascularization and perfusion recovery in peripheral arterial disease.

**Methods and Results:**

We first determined that 4 weeks of precursory treadmill exercise improved perfusion recovery on days 7, 14 and 21 after unilateral femoral artery ligation (FAL) but had no effect immediately after ligation. Then, local muscle delivery of Exe-Exo promotes arteriogenesis, angiogenesis and perfusion recovery, which could be abolished by GW4869, a well-recognized pharmacological agent inhibiting exosome release. This suggests that Exe-Exo mediated exercise-induced revascularization. *In vitro*, Exe-Exo enhanced endothelial cell proliferation, migration and tube formation. In addition, we identified miR-125a-5p as a novel exerkine through exosomal miRNA sequencing and RT-qPCR validation. Inhibition of miR-125a-5p abrogated the beneficial effects of Exe-Exo both *in vivo* and *in vitro*. Mechanistically, these exercise-afforded benefits were attributed to the exosomal miR-125a-5p downregulation of ECE1 expression and the subsequent activation of the AKT/eNOS downstream signaling pathway. Specifically, skeletal muscle may be a major tissue source of exercise-induced exosomal miR-125a-5p *via* fluorescence *in situ* hybridization.

**Conclusions:**

Endogenous circulating exosomal miR-125a-5p promotes exercise-induced revascularization *via* targeting ECE1 and activating AKT/eNOS downstream signaling pathway. Identify exosomal miR-125a-5p as a novel exerkine, and highlight its potential therapeutic role in the prevention and treatment of peripheral arterial disease.

## Graphical Abstract

Proposed mechanisms by which exercise-induced circulating exosomes promote arteriogenesis and angiogenesis after lower limb ischemia. Four weeks of exercise training mainly increased miR-125a-5p expression in adductor muscles and cargoes into exercise-derived exosomes. These miR-125a-5p-enriched exosomes were delivered to endothelial cells and enhanced blood perfusion recovery after femoral artery ligation by targeting ECE1 and activating the AKT/eNOS signaling pathway. 
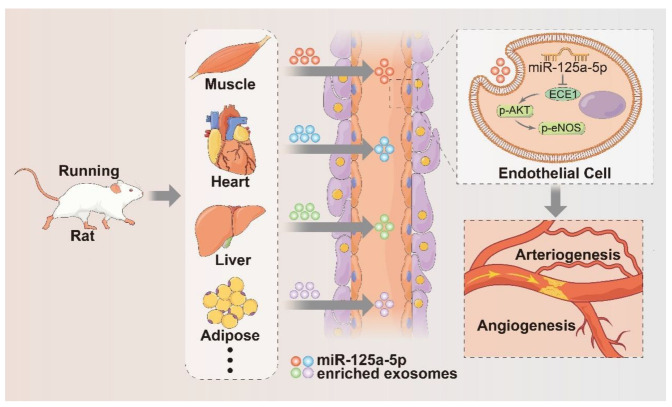

## Introduction

The beneficial effects of regular physical exercise in preventing and treating ischemic diseases have long been appreciated (1). Exercise prior to stroke exerts endogenous neuroprotection by enhancing angiogenesis (2). Exercise preconditioning induces potential cardioprotective effects including anti-atherosclerosis, anti-thrombosis, anti-ischemia and anti-arrhythmia (3). To date, catheter- and surgical-based revascularization methods are currently the preferred therapies for patients with peripheral arterial disease (PAD) or coronary artery disease (CAD). However, some patients are not suitable for receiving interventional treatment (4). Therefore, it is important and urgent to seek novel therapeutic approaches to induce endogenous revascularization, which comprises both angiogenesis and arteriogenesis in adults (5, 6). Arteriogenesis involves collateral growth and remodeling of pre-existing arterioles, while angiogenesis is the process of sprouting new capillaries from existing vessels (7, 8). Moreover, the mechanisms responsible for the beneficial effects of physical exercise on arteriogenesis and angiogenesis mainly focused on endothelial function and nitric oxide bioavailability (9, 10).

Exosomes are small, single-membrane, naturally derived 30–150 nm nanovesicles that play essential roles in cell-to-cell and/or cell-to-tissue communications through the efficient delivery of selected bioactive molecules, including nucleic acids (such as mRNA and miRNA), proteins and lipids (11). Multiple studies have shown that the amount, composition and molecular profile of exosomes are reflected and affected by physiological and pathophysiological conditions, among which exercise, as an important factor changing the overall state of the body, plays a crucial role in the formation and release of exosomes (12, 13). Carsten et al. demonstrated that a single bout of exhaustive exercise induces rapid release of exosomes into circulation but is cleared during the early recovery period after cycling in humans (14). Intriguingly, exercise can trigger the release of various “exerkines” into the circulation that can be encapsulated within exosomes (15). Furthermore, it has been reported that exosomes released from cardiomyocytes (16), endothelial cells (17), cardiac fibroblasts (18) and cardiac progenitor cells (19) could exert cardioprotection *via* the transmission of miRNAs. Importantly, it was recently reported that long-term exercise-derived circulating exosomal miR-342-5p acts as a novel cardioprotective exerkine against myocardial ischemia/reperfusion injury in rats (20), providing compelling evidence that circulating exosomes can deliver endogenous protective signals to distal organs, even under physiological conditions. Therefore, we hypothesized that circulating exosomes enriched with certain miRNAs may be a key regulator of precursory exercise-induced endogenous revascularization in ischemic cardiovascular diseases.

In the current study, we used a rat hindlimb ischemia model to investigate the effects of exercise on arteriogenesis, angiogenesis and recovery perfusion. We demonstrated a novel endogenous protective mechanism by which prophylactic exercise promotes revascularization mediated by circulating exosomes enriched with miR-125a-5p.

## Materials and methods

### Animals

All animal care protocols and surgical procedures were reviewed and approved by the Animal Care and Use Committee of the Department of Laboratory Animals, Central South University (2019030404). Eight-week-old male Sprague–Dawley (SD) rats (Slac Laboratory, Shanghai, China) were maintained in accordance with the guidelines from Directive 2010/63/EU of the European Parliament on the protection of animals used for scientific purposes. The SD rats were anaesthetized with a 2% isoflurane/oxygen mixture when subjected to any invasive operations under sterile conditions. Buprenorphine (1 mg/kg) was administered subcutaneously for postsurgical analgesia. For the isolation of the blood and tissue, rats were euthanized with carbon dioxide (CO2) until respiration, and heart was completely stopped.

### Rat model of hindlimb ischemia

We performed femoral artery ligature (FAL) according to previous studies with slight modifications (21, 22). Briefly, a vertical longitudinal incision was made in the unilateral hindlimb, and the femoral artery was gently separated from the femoral vein and nerve. Then both the saphenous artery and the superficial caudal epigastric artery were ligated with 5-0 sutures. The wound was irrigated with saline and closed with 3-0 sutures. Antibiotics (cefazolin, i.m.) and analgesics (buprenorphine, s.c.) were administered.

### Long-term treadmill exercise training

Exercise was performed on a treadmill that was specially designed for rats. The treadmill had different lanes to serve as corridors for each animal and had a grid in the back that administered a small electric shock on contact to ensure that the animals ran effectively. The animals were randomly assigned to sedentary (Sed) or exercise (Exe) groups. The Exe groups were subjected to moderate-intensity interval treadmill training for 4 weeks. In the first week: 10 m/min for 20 min on the first day. Then on the following days, the speed was increased by 5 m/min and the time was increased by 10 min every day, until 30 m/min for 60 min on the fifth day. Thereafter, the animals were trained at this level for 5 days per week for the following 3 weeks. The sed groups were exposed to the same noise and vibration of the environment (23, 24). FAL surgery was performed 24 h after the last training day.

### Statistical analysis

All quantitative results are expressed as the mean ± SEM. An assumption of normality of data was tested using a normality and lognormality test. The Brown-Forsythe test was used for testing the equality of variances. Comparisons between the two groups were analyzed by unpaired Student’s *t* test. Multiple comparisons over two groups were analyzed with one or two-way ANOVA followed by Tukey’s *post hoc* tests. If data did not pass normality test, using the Mann–Whitney *U* for 2 groups or Kruskal–Wallis tests for multiple group comparisons, followed by Tukey’s *post hoc* test. All experiments were repeated at least 3 times. The figure plotting and statistical analysis was conducted using GraphPad Prism 9 software and a two-sided *p* < 0.05 was considered statistically significant.

For further details regarding materials and methods, see the Supplementary Material.

## Results

### Prophylactic exercise increases perfusion recovery after hindlimb ischemia in rats

To determine the role of prophylactic exercise in hindlimb perfusion recovery, the rats were subjected to treadmill training for 4 weeks followed by unilateral femoral artery ligation (Supplementary Figure 1A). Hindlimb perfusion restoration was measured *via* laser-Doppler imaging. As presented in Supplementary Figures 1B,C, no significant difference was observed immediately after the surgery compared with sedentary controls, indicating that exercise does not influence baseline blood perfusion. However, exercised rats showed an increased perfusion recovery on days 7, 14 and 21 after femoral artery ligation, demonstrating that 4 weeks of exercise training improves perfusion recovery after hindlimb ischemia.

### Characterization and *in vitro* functional validation of circulating exosomes from sedentary and exercised rats

**FIGURE 1:**
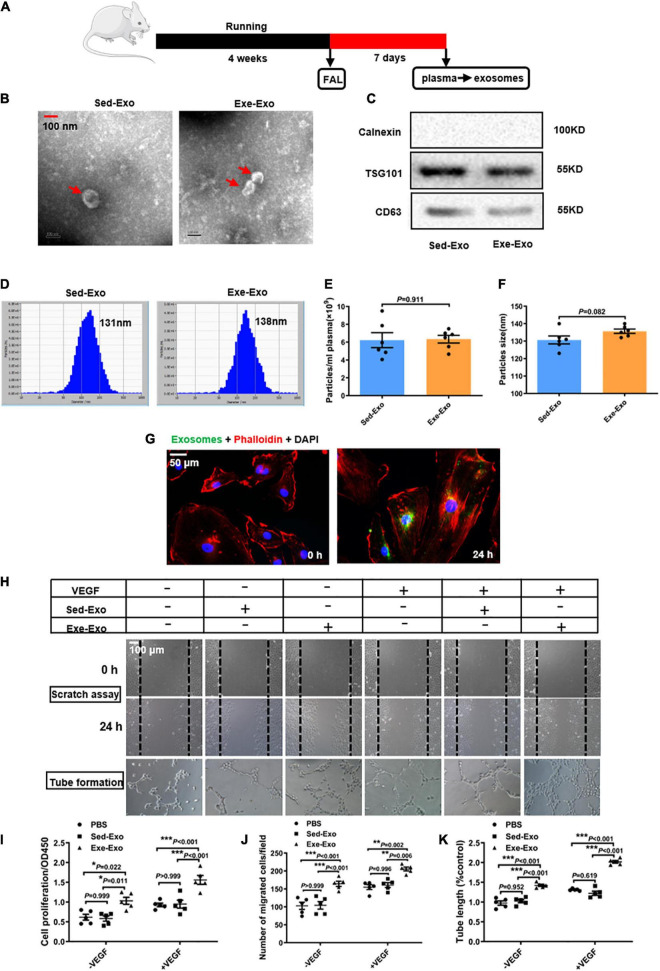
Characterization and *in vitro* functional validation of circulating exosomes from sedentary and exercised rats. **(A)** Schematic diagram of plasma-derived exosome acquisition. Exosomes were isolated from the plasma of rats 7 days after FAL surgery following 4 weeks of running exercise or sedentariness. **(B)** Representative transmission electron micrographs of Sed-Exo and Exe-Exo. Scale bar = 100 nm. Arrows, representative Exosomes. **(C)** Representative immunoblots of exosomal marker proteins (TSG101 and CD63) and negative control protein (calnexin) in Sed-Exo and Exe-Exo. **(D–F)** Representative results of nanoparticle tracking analysis of Sed-Exo and Exe-Exo. Quantitative analysis of the average concentration **(E)** and size distribution **(F). (G)** Internalization of PKH67-labeled exosomes. HUVECs were incubated with exosomes for 0 or 24 h. Representative images showing the internalization of PKH67-labeled exosomes (green) by HUVECs. Red, Phalloidin, used for staining actin filaments. Green, PKH67-labeled exosomes **(H–K)**. Cultured HUVECs were incubated with Sed-Exo (50 μg/ml) or Exe-Exo (50 μg/ml) for 48 h, followed by starvation for 6 h and stimulation with VEGF165 (100 ng/ml) for 20 min. Cells proliferation was determined by CCK8 **(I).** Cell migration was determined by scratch assay, representative images **(H)** and quantitative analysis **(J).** Representative images **(H)** and a quantitative analysis **(K)** of tube formation. FAL, femoral artery ligation. Sed-Exo, exosome purified from the plasma of sedentary rats. Exe-Exo, exosome purified from the plasma of exercised rats. *P < 0.05, **P < 0.01, ***P < 0.001. Each experiment was repeated 5 times. Scale bar = 50 or 100 μm as presented in the above images. Data are means ± SEM.

Exosomes were isolated from the plasma of rats 7 days *via* ultracentrifugation after left femoral artery ligation following 4 weeks of running training (Exe-Exo) or sedentariness (Sed-Exo) (Figure 1A). A transmission electron microscopy analysis revealed cup-shaped particles with diameters of 30–150 nm (Figure 1B). Immunoblotting was used to identify the exosomal markers TSG101 and CD63. The purity of the isolated exosomes was confirmed *via* calnexin which is absent in exosomes (Figure 1C). A nanoparticle tracking analysis exhibited no significant differences in the plasma concentration (6.225 ± 2.06 × 10^9^ ml^–1^ vs. 6.333 ± 1.06 × 10^9^ ml^–1^) or size distribution (130.667 ± 5.57 nm vs. 135.667 ± 3.01 nm) between Sed-Exo and Exe-Exo (Figures 1D–F). We incubated HUVECs with PKH67-labeled exosomes for 24 h and observed that the exosomes were internalized, as presented by immunofluorescence (Figure 1G). Interestingly, HUVECs preincubated with Exe-Exo, but not Sed-Exo for 48 h showed a higher proliferation (Figure 1I), migration (Figures 1H,J) and tube formation (Figures 1H,K) abilities at baseline or stimulated by VEGF *in vitro*.

### Local muscle delivery of exercise-derived circulating exosomes promotes arteriogenesis, angiogenesis and perfusion recovery, which could be abolished by GW4869

**FIGURE 2:**
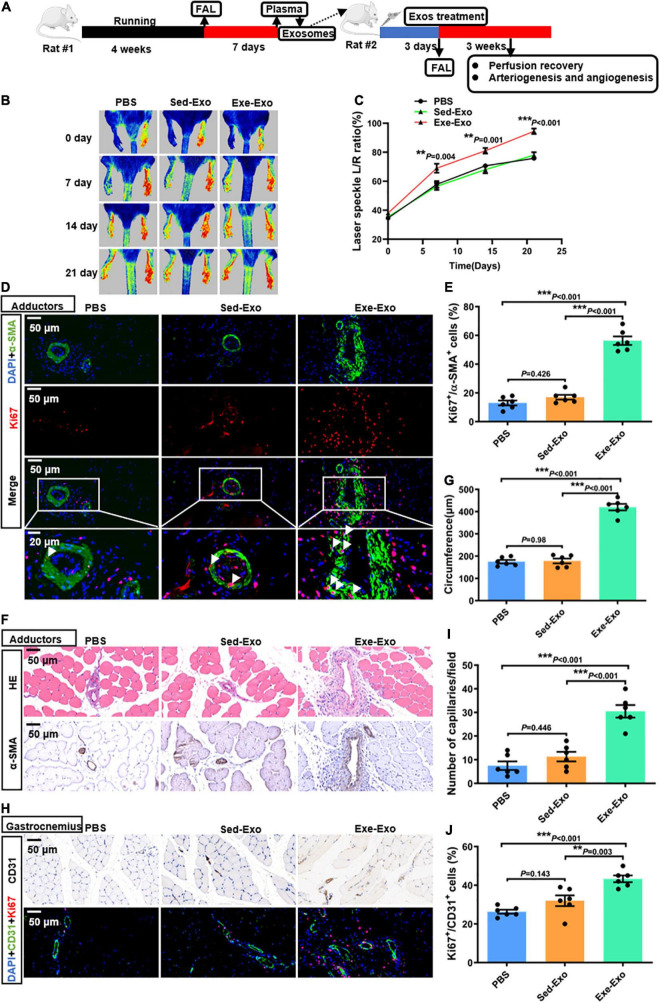
Local muscle delivery of exercise-derived circulating exosomes promoted arteriogenesis, angiogenesis and perfusion recovery. **(A)** Rats were subjected to 4 weeks of treadmill training or kept as sedentary controls followed by FAL surgery. Then, equivalent quantities of exosomes (1 mg, mixed with pluronic gel) isolated from the plasma of rats (Exe or Sed) 7 days post-FAL were injected into the left adductor muscles of the second batch of healthy adult rats. Subsequently, all the second batch rats were exposed to FAL surgery, and perfusion recovery, and arteriogenesis and angiogenesis were measured. **(B,C)** Representative laser speckle perfusion images **(B)** and quantitative analysis **(C)** of the ratios of left to right (L/R) hindlimb perfusion among PBS-, Sed-Exo- and Exe-Exo- treated rats at the indicated times after FAL. **(D,E)** Representative images of immunofluorescence double staining **(D)** and quantitative analysis **(E)** of the cross-sections of adductor muscles 7 days after the surgery. The ratio of Ki67-positive cells to SMCs in each field was quantified. Red, Ki67. Green, α-SMA. Blue, 4’,6-diamidino-2-phenylindole (DAPI). **(F,G)** Representative images **(F)** and a quantitative analysis **(G)** of HE staining and immunohistochemistry of α-SMA in cross-sections of the adductor muscles from PBS-, Sed-Exo- and Exe-Exo- treated rats 7 days after surgery. **(H–J)** Representative images **(H)** and a quantitative analysis **(I,J)** of immunofluorescence double staining and CD31 immunohistochemistry in cross-sections of the gastrocnemius muscles from PBS-, Sed-Exo- and Exe-Exo- treated rats 7 days after surgery. The ratio of Ki67-positive cells to total ECs in each field was quantified. Red, Ki67. Green, CD31. Blue, DAPI. Arrows, representative Ki67 positive SMCs. *N* = 6 per group. **P < 0.01, ***P < 0.001. FAL indicates femoral artery ligation. Scale bar = 20 or 50 μm as presented in the above images. Data are means ± SEM.

*In vitro* data have indicated that Exe-Exo promotes HUVECs proliferation, migration and tube formation, so we next confirmed these results *in vivo*. Therefore, Exe-Exo and Sed-Exo were isolated from the rats and locally injected into adductors 3 days before left femoral artery ligation (Figure 2A). Firstly, we found that adductor muscles injected with Exe-Exo revealed an increased miR-125a-5p expression than other groups, suggesting a successful exosomes injection indirectly (Supplementary Figure 2A). Moreover, to determine the localization of exosomes after injection in adductor muscle, we designed an experiment. 1 mg of Sed-Exo or Exe-Exo mixed with pluronic gel was injected into the adductor muscle by five separate injections. We found that PKH67 fluorescence was observed within adductor muscle 24 h after intramuscular injection of PKH67-labeled exosomes (Supplementary Figure 2B), suggesting an efficient *in vivo* uptake of the exosomes by adductor muscle directly. Notably, as shown in Figures 2B,C, we observed an increased perfusion recovery in the ischemic limb with Exe-Exo treatment at days 7,14 and 21. We next determined whether Exe-Exo influences revascularization. We found that adductor muscles injected with Exe-Exo revealed an increased number of Ki67-positive smooth muscle cells (SMCs) in collateral arteries compared with those injected with Sed-Exo and PBS by double staining for Ki67 and α-SMA immunofluorescence (Figures 2D,E), which was accompanied by an increased luminal circumference of these blood vessels (Figures 2F,G). Moreover, Exe-Exo administration also increased the capillary density and Ki67-positive endothelial cells (ECs) in the gastrocnemius muscle (Figures 2H–J). These data suggest that Exe-Exo remarkably promotes arteriogenesis and angiogenesis *in vivo*.

To evaluate whether these beneficial effects could be abolished by inhibiting exercise-induced exosome generation, we used a pharmacological agent, GW4869, which is well recognized to inhibit the ceramide-mediated inward budding of multivesicular bodies (MVBs) and release of mature exosomes from MVBs (16, 25). Rats were injected intraperitoneally with either DMSO or GW4869 (2 μg/g) every other day during the entire observation period, followed by exercise training and femoral artery ligation (Supplementary Figure 3A). We noticed that GW4869 treatment significantly decreased the exosome levels in plasma by 36%, compared to levels collected from control rats (Supplementary Figure 3B). As expected, the pre-treatment of rats with GW4869 evoked an impairment of arteriogenesis and angiogenesis, as evidenced by a significant decrease in hindlimb blood perfusion, numbers of Ki67-positive SMCs or ECs, collateral circumference and capillary density, as compared to the controls (Supplementary Figures 3C–K). Collectively, these observations suggest that the blockade of exosome generation with GW4869 diminishes functional blood vessel formation following hindlimb ischemia injury.

### miR-125a-5p plays a crucial role in Exe-Exo-induced HUVEC proliferation, migration and tube formation

**FIGURE 3:**
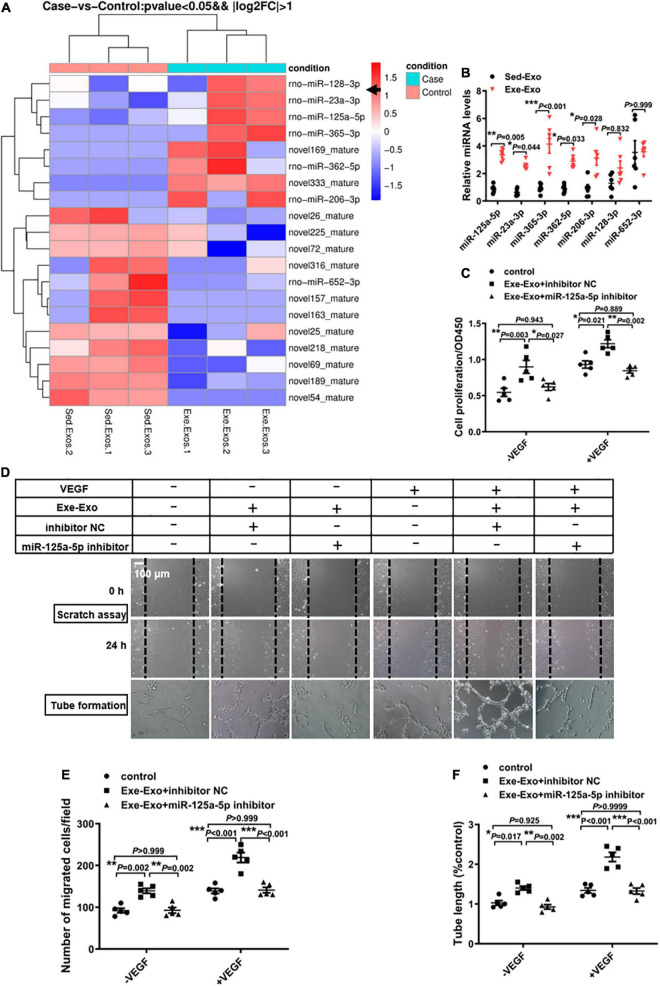
miR-125a-5p is essential in Exe-Exo-induced HUVEC proliferation, migration and tube formation. **(A)** Exosomal miRNA sequencing assays were performed in Exe-exo and Sed-exo. Heat map showing the differential expression of miRNAs between the 2 groups and with a level change of greater than twofold (*p* < 0.05). Sed-Exo, exosomes purified from the plasma of sedentary rats. Exe-Exo, exosomes purified from the plasma of exercised rats. *N* = 3 per group. **(B)** RT-qPCR analysis of the 7 differentially expressed known miRNAs in Sed-Exo and Exe-Exo. Data are normalized to spiked cel-miR-39 (*N* = 6). **(C–F)** Cultured HUVECs were transfected with an miR-125a-5p inhibitor (100 nM) or inhibitor NC (100 nM) and incubated with Exe-Exo (50 μg/ml) for 48 h, the followed by starvation for 6 h and stimulation with VEGF165 (100 ng/ml) for 20 min. Cell proliferation was determined by CCK8 **(C).** Cell migration was determined by scratch assay, representative images **(D)** and a quantitative analysis **(E).** Representative images **(D)** and a quantitative analysis **(F)** of tube formation. Each experiment was repeated 5 times. *P < 0.05, **P < 0.01, ***P < 0.001. Scale bar = 100 μm as presented in the above images. Data are means ± SEM.

Exosomes regulate a large number of physiological activities *via* exosomal miRNAs (26). Thus, we explored whether or not exercise induced exosomes promote revascularization through exosomal miRNAs. First, a miRNA deep sequence analysis (229 known rat miRNAs and 335 predicted novel miRNAs) was conducted to identify the differentially abundant miRNAs between Exe-Exo and Sed-Exo. A total of 20 differentially expressed miRNAs (fold change > 2.0; *P* < 0.05; Figure 3A and Supplementary Table 1) were detected, and all the known miRNAs were further validated by an RT-qPCR analysis. Among the 7 known miRNAs, 5 miRNAs (rno-miR-125a-5p, rno-miR-23a-3p, rno-miR-365-3p, rno-miR-362-5p and rno-miR-206-3p) were significantly upregulated in Exe-Exo (Figure 3B). Among them, we focused on miR-125a-5p, the most abundant miRNA in plasma-derived exosomes released by exercise or sedentary rats (Supplementary Table 1). Moreover, a previous study reported that mesenchymal stem cell-derived exosomes can transfer miR-125a to endothelial cells and promote angiogenesis by repressing DLL4 (27).

To determine whether miR-125a-5p is involved in Exe-Exo-mediated EC proliferation and migration, loss-of-function studies were performed. We found that inhibition of miR-125a-5p with an inhibitor markedly decreased the promotion effect on EC proliferation (Figure 3C), migration (Figures 3D,E) and tube formation (Figures 3D,F) of exercise-derived circulating exosomes, both on quiescent ECs and stimulation with VEGF.

### miR-125a-5p is indispensable in exercise-induced arteriogenesis and angiogenesis *in vivo*

**FIGURE 4:**
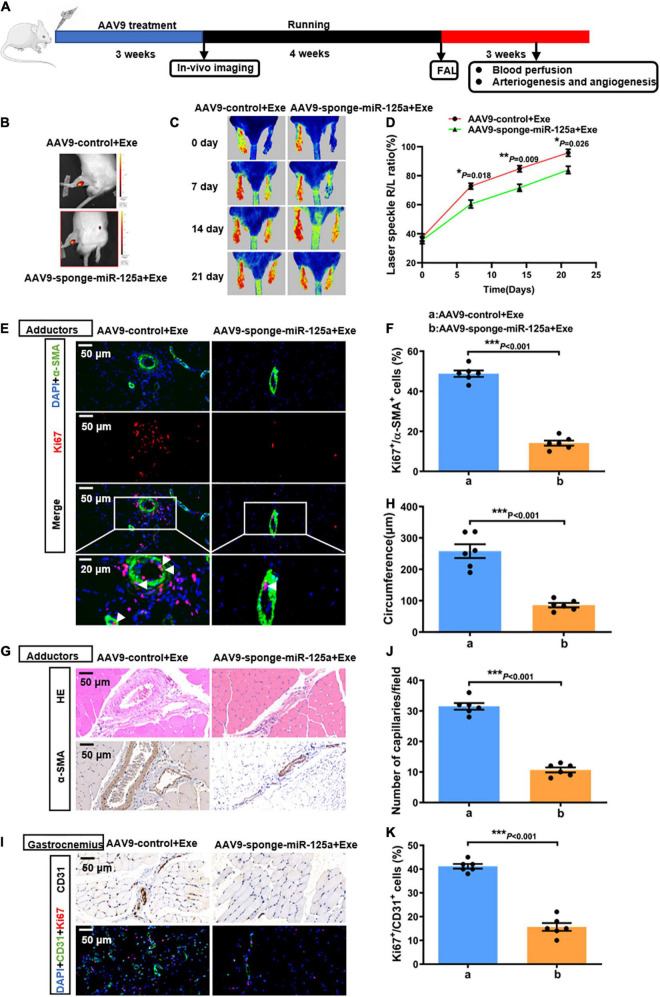
miR-125a-5p was indispensable in exercise-induced arteriogenesis and angiogenesis *in vivo*. **(A)** Rats were randomly assigned to receive adductor injection *in situ* of either AAV9-sponge-miR-125a-5p-mCherry or AAV9-control-mCherry (1*10^12^ vg/ml, 15 μl/point, 5 points) per animal. After 3 weeks, the rats were subjected to *in vivo* imaging. Then the rats were subjected to 4 weeks of treadmill exercise training followed by FAL surgery, and hindlimb perfusion recovery was measured postoperatively on selected days *via* laser Doppler. **(B)** Representative *in vivo* images. **(C,D)** Representative laser speckle perfusion images **(C)** and a quantitative analysis **(D)** of the ratios of right to left (R/L) hindlimb perfusion among AAV9-control + Exe- and AAV9-sponge-miR-125a-5p + Exe- treated rats at indicated times after FAL. **(E,F)** Representative images of immunofluorescence double staining **(E)** and quantitative analysis **(F)** of the cross-sections of adductor muscles 7 days after the surgery. Red, Ki67. Green, α-SMA. Blue, DAPI. **(G,H)** Representative images **(G)** and quantitative analysis **(H)** of HE staining and immunohistochemistry of α-SMA in cross-sections of the adductor muscles from AAV9-control + Exe- and AAV9-sponge-miR-125a-5p + Exe treated rats 7 days after surgery. **(I-K)** Representative images **(I)** and quantitative analysis **(J,K)** of immunofluorescence double staining and CD31 immunohistochemistry in cross-sections of the gastrocnemius muscles from AAV9-control + Exe- and AAV9-sponge-miR-125a-5p + Exe- treated rats 7 days after surgery. The ratio of Ki67-positive cells to the total ECs in each field was quantified. Red, Ki67. Green, CD31. Blue, DAPI. Arrows, representative Ki67 positive SMCs. *N* = 6 per group. *P < 0.05, **P < 0.01, ***P < 0.001. AAV9-control + Exe vs. AAV9-sponge-miR-125a-5p + Exe. FAL indicates femoral artery ligation. Scale bar = 20 or 50 μm, as presented in the above images. Data are means ± SEM.

To elucidate the role of miR-125a-5p in exercise-induced endogenous revascularization *in vivo*, we used AAV9 carrying a specific sequence targeting miR-125a-5p to achieve local neutralization of miR-125a-5p in rat hindlimbs *in vivo*. Rats received adductor injections of either AAV9-sponge-miR-125a-5p or AAV9-control *in situ*. mCherry, an indicator for the AAV vector, was detectable *via in vivo* imaging 3 weeks after injection (Figure 4B). Then, the rats were subjected to 4 weeks of treadmill running followed by an FAL surgery (Figure 4A). As depicted in Supplementary Figure 4A, we confirmed that AAV9-sponge-miR-125a-5p efficiently reduced the miR-125a-5p expression levels in adductor muscles compared with AAV9-control. Importantly, perfusion recovery was significantly blunted at days 7,14 and 21 after the surgery *via* AAV9-sponge-miR-125a-5p treatment (Figures 4C,D). Similarly, a decreased number of Ki67-positive SMCs or ECs, collateral circumference and capillary density were observed, compared to AAV9-control which received exercise training for 4 weeks (Figures 4E–K). Together, these alterations induced by exercise were bypassed by AAV9-sponge-miR-125a-5p pre-treatment, indicating that miR-125a-5p plays a key role in exercise induced revascularization *in vivo*.

### miR-125a-5p promotes proliferation, migration and tube formation of HUVEC by targeting endothelin converting enzyme 1 (ECE1)

**FIGURE 5:**
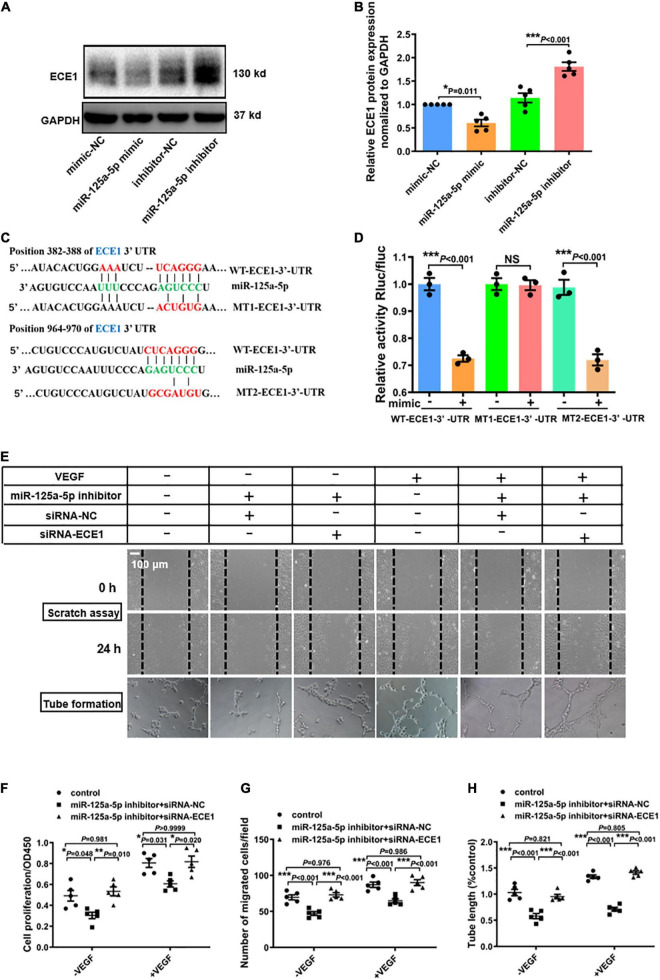
miR-125a-5p promotes the proliferation, migration and tube formation of HUVECs by targeting ECE1. **(A,B)** HUVECs were transfected with miR-125a-5p mimic (50 nM) or miR-125a-5p inhibitor (100 nM) and the corresponding negative controls for 48 h. ECE1 protein was determined *via* western blotting. **P < 0.01 vs. the corresponding negative control. **(C)** TargetScan prediction of miR-125a-5p targeting the 3′-UTR of ECE1 mRNA. **(D)** Plasmids for luciferase reporter construction containing the full-length wild-type 3′-UTR of ECE1 mRNA (WT-3′-UTR) and mutated 3′-UTR of ECE1 mRNA (MT1-3′-UTR and MT2-3′-UTR) were generated, and 293T cells were co-transfected with an miR-125a-5p mimic or negative control. The luciferase activity in the cells was assayed. *N* = 3 per group. **P < 0.01. **(E–G)** HUVECs were co-transfected with an miR-125a-5p inhibitor or inhibitor NC and siRNA-ECE1 or siRNA-NC for 48 h. Then the cells were treated with or without starvation for 6 h followed by stimulation with VEGF165 (100 ng/ml) for 20 min. Cell proliferation **(F)** and migration **(E,G)** were determined *via* CCK-8 and scratch assay respectively. Representative images **(E)** and a quantitative analysis **(H)** of tube formation. *P < 0.05, **P < 0.01, ***P < 0.001. Each experiment was repeated 3–5 times. Scale bar = 100 μm, as presented in the above images. The data are means ± SEM.

Based on the *in vitro* and *in vivo* data, we hypothesized that miR-125a-5p targets critical components in EC activation. Accordingly, we scanned predicted target genes of miR-125a-5p obtained from TargetScan and miRanda. One interesting candidate was ECE1, which contains two predicted miR-125a-5p-binding sites in the 3′-UTR (Figure 5C) and exhibited an important role in regulating vascular tone, growth and development. Notably, overexpression or inhibition of miR-125a-5p by transfecting HUVEC with a mimic or inhibitor significantly reduced or increased the protein levels of ECE1, respectively (Figures 5A,B). Furthermore, the expression of ECE1 in adductor muscles was increased by AAV9-sponge-miR-125a-5p treatment (Supplementary Figures 4B,C). These observations indicated that miR-125a-5p can regulate the expression of ECE1 *in vitro* and *in vivo*.

To verify whether miR-125a-5p can directly target ECE1 through the 3′-UTR interaction, we generated three luciferase reporter plasmids with full-length wild-type ECE1 3′-UTR (WT-3′-UTR) and mutated ECE1 3′-UTRs (MT1-3′-UTR, MT2-3′-UTR) and detected the luciferase activity in 293T cells. The miR-125a-5p mimic significantly suppressed the luciferase activity fused with the WT-3′-UTR or MT2-3′-UTR of ECE1, but exerted no effect on the MT1-3′-UTR, suggesting that ECE1 was the target of miR-125a-5p (Figures 5C,D).

Additionally, to further confirm that ECE1 is a functional target gene of miR-125a-5p, gain- and loss-of-function studies were performed. As presented in Figures 5E–G, siRNA-ECE1 rescued the inhibitory effects of the miR-125a-5p inhibitor on EC proliferation, migration and angiogenesis, as determined by CCK-8 and scratch assays and tube formation, respectively (Figures 5E–H). Collectively, these data provide compelling evidence that miR-125a-5p regulates ECs functions by targeting ECE1.

### Exercise-derived circulating exosomes activate the AKT/eNOS signaling pathway *via* miR-125a-5p targeting ECE1

**FIGURE 6:**
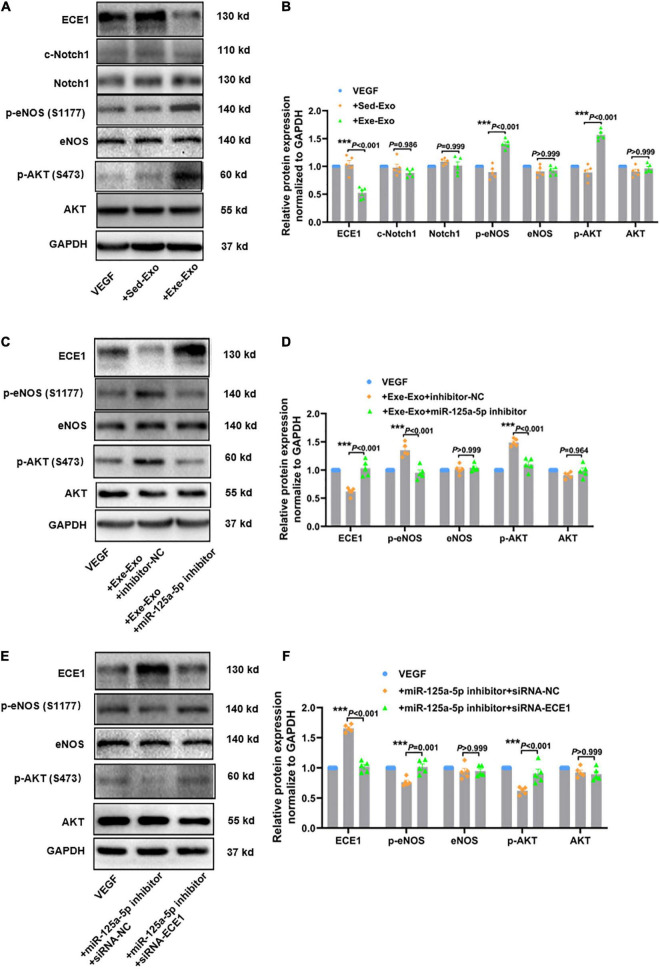
Exercise-derived circulating exosomes activate the AKT/eNOS signaling pathway *via* miR-125a-5p targeting ECE1. The cells were incubated with Sed-Exo (50 μg/ml) or Exe-Exo (50 μg/ml) for 48 h, starved for 6 h and stimulated with VEGF165 (100 ng/ml) for 20 min. Exe-Exo downregulated ECE1 expression and activated AKT and eNOS phosphorylation but had no effect on Notch1 cleavage. Representative blots **(A)** and quantitative analysis **(B)** of ECE1, Notch1, c-Notch1, AKT, p-AKT, eNOS and p-eNOS protein expression determined *via* western blotting. Cultured HUVECs were incubated with Exe-Exo (50 μg/ml) and transfected with an miR-125a-5p inhibitor (100 nM) or inhibitor NC (100 nM) for 48 h, followed by starvation for 6 h and stimulation with VEGF165 (100 ng/ml) for 20 min. Representative blots **(C)** and quantified data analysis **(D)** of ECE1, AKT, p-AKT, eNOS and p-eNOS protein expression determined by western blotting. HUVECs were co-transfected with miR-125a-5p inhibitor and siRNA-ECE1 or siRNA-NC for 48 h. Then the cells were treated with or without starvation for 6 h followed by stimulation with VEGF165 (100 ng/ml) for 20 min. Representative blots **(E)** and quantified data **(F)** of ECE1, AKT, p-AKT, eNOS and p-eNOS protein expression determined *via* western blotting. ***P < 0.001. Each experiment was repeated 5 times. The data are means ± SEM.

It has been well documented that AKT/eNOS and Notch1 are the key signaling molecules regulating vessel growth (28). Thus, we reasoned that exercise-derived circulating exosomes may promote revascularization by activating AKT/eNOS phosphorylation or Notch1 activation. To test this hypothesis, we incubated HUVECs with Sed-Exo or Exe-Exo for 48 h, followed by starvation for 6 h and stimulation with VEGF for 20 min, and detected protein expression *via* western blot. The phosphorylation of Akt (Ser473) and the downstream effector eNOS (S1177) were elevated by Exe-Exo, concomitantly with reduced ECE1 expression, while the cleavage of Notch1 was not significantly changed (Figures 6A,B). These results indicate that the AKT/eNOS pathway rather than Notch1 plays a critical role in Exe-Exo-mediated angiogenesis and arteriogenesis.

To further explore its specific mechanism, we designed a series of loss- and gain-function experiments. As shown in Supplementary Figures 5A,B, an enhanced AKT/eNOS phosphorylation and a reduced ECE1 expression were observed upon overexpression of miR-125a-5p, whereas the miR-125a-5p inhibitor reversed these effects. Subsequently, we elucidated whether miR-125a-5p could mediate the effect of Exe-Exo on HUVECs. We incubated HUVECs with Exe-Exo and transfected them with an miR-125a-5p inhibitor and found that downregulated miR-125a-5p expression compromised the beneficial effects of Exe-Exo (Figures 6C,D). Similarly, as presented in Figures 6E,F, downregulating the ECE1 protein expression by siRNA negated the inactivation of the AKT/eNOS signaling pathway. Together, these findings supported the notion that exercise-derived circulating exosomes activate the AKT/eNOS signaling pathway *via* miR-125a-5p targeting ECE1.

### Skeletal muscle might be a primary origin of exercise-induced exosomal miR-125a-5p

Physical exercise stimulates organs, mainly skeletal muscles, to release a broad range of molecules, dubbed exerkines. Among them, miRNAs loaded in exosomes have the potential to play a critical role in translating exercise into health (29). As shown in Supplementary Figure 6D, the change in miR-125a-5p expression in adductor muscle was not statistically significant, and a trend toward increased expression was observed at the indicated times after FAL by RT-qPCR. Nevertheless, miR-125a-5p increased sharply by approximately 5.3-fold in the group at 7 days post-FAL following 4 weeks of exercise (Exe-7 day), which is close to the expression change of the exercise itself compared with the sedentary control group, indicating that the increase in miR-125a-5p was mainly induced by exercise (Supplementary Figures 6A–C). Moreover, we examined the expression of miR-125a-5p in various organs and tissues *via* FISH, such as skeletal muscle, heart, liver, kidney, aorta and adipose tissue. Apparently, miR-125a-5p is abundantly expressed in skeletal muscle and is significantly increased in the exercise group compared with the sedentary group. However, it was either relatively low or undetectable and had no significant differences in other tissues.

Because circulating exosomes are mainly released from endothelial and blood cells (30), we counted endothelial-, platelet-, and other cell-derived exosomes using nanoflow cytometry to explore whether there were differences in the cellular origins between the sedentary and exercise groups. Unexpectedly, no significant difference was found at the cellular source level (Supplementary Figure 7).

### Ischemic and healthy lower limbs showed the same tendency to respond to miR-125a-5p

Moving from basic to the clinic, ischemic and healthy lower limbs also respond differently. Therefore, we designed experiments to determine what extent ischemic limbs respond differently to miR-125a-5p compared to healthy lower limbs. We found that in the sham groups, there was no statistical difference in lower limb blood flow recovery between the two intervention groups, but in the FAL groups, miR-125a-5p significantly promoted perfusion recovery (Supplementary Figures 8A,B). However, both in sham and FAL groups, increase of angiogenesis were observed under agomir-125a-5p injection (Supplementary Figures 8C,D), which is consistent with the effect of miR-125a-5p on endothelial cells under baseline and VEGF stimulation *in vitro*.

## Discussion

In this paper, we report on the exciting new observation that endogenous circulating exosomal miR-125a-5p is a novel exerkine promoting precursory exercise-induced arteriogenesis, angiogenesis and perfusion recovery *via* targeting ECE1 and activating AKT/eNOS signaling pathway in endothelial cells (Graphical Abstract).

Of all the deaths caused by major non-communicable diseases, a considerable share of up to one-tenth result from physical inactivity (31). However, the underlying mechanisms of exercise-conferred revascularization have not been fully elucidated. Notably, FAL animal models and patients with PAD or CAD usually present similar neovascularization patterns, with established arteriogenesis adjacent to the occlusion site and enhanced angiogenesis in the distal ischemic tissue (8, 32). Moreover, Stephan et al. reported that 3 weeks of exercise training before FAL surgery significantly increased perfusion in a murine hindlimb model but had no more effect when continuing to treat the mouse with exercise after the surgery (10). This could cause inevitable pain to animals when subjected to continuous running exercise after FAL surgery. Therefore, in the present study we adopted an exercise preconditioning model in which 4 weeks of treadmill running followed by unilateral FAL was performed and observed that prophylactic exercise could exert a sustained improvement in perfusion recovery from 7 to 21 days after FAL.

It has been well recognized that certain RNA species, metabolites and peptides (collectively termed “exerkines”) are released in response to exercise that could facilitate multisystemic benefits and could serve as potential therapeutic targets (33). Specifically, interleukin 6 (IL-6) has been identified as the first muscle-derived exerkine and improves glucose and fat metabolism by increasing glucose uptake and fatty acid oxidation (34, 35). Recently, it was discovered that exosomes could transfer exerkines from cell to cell to mediate exercise-induced beneficial effects in an autocrine, paracrine, and endocrine manner (36). However, the type, duration, and intensity of exercise can modify the number, content and cell origin of exosome secretion (37). Previous studies have reported that a single bout of acute exercise provokes the rapid release of exosomes into circulation, but the circulating exosome level returns to baseline within 24 h after the last training session (14, 38). In the current study, we discovered that the exosome level was unchanged in the groups 7 days after left femoral artery ligation following 4 weeks of running training or sedentariness. In particular, exosomes isolated from the plasma of exercised rats could be internalized by EC and induce neovascularization, as evidenced by increased EC proliferation, migration and tube formation *in vitro*. Moreover, it was further confirmed that exosomes increased perfusion recovery, levels of arteriogenesis and angiogenesis in the ischemic limb. Alternatively, the blockade of exosome generation with GW4869 exerted the opposite effects. Collectively, these results indicated that the change in the contents of circulating exosomes, but not the alteration in the counts, might be responsible for the benefits of exercise.

Exosomes are thought to play crucial roles in intercellular communication by transporting their encircled contents, such as miRNAs, mRNA and proteins, through systemic circulation. Importantly, exosomes could protect their cargoes from degradation (39). To our knowledge, exosomal miRNAs have recently arisen as the best known and most studied non-coding RNA, and represent a promising alternative therapy against ischemic diseases (8). For instance, CD34^+^ stem cell-derived exosomal miR-126-3p mediates tissue repair of ischemic hindlimbs *via* beneficial angiogenesis (40). Using miRNA deep sequencing and RT-qPCR verification, we discovered that 5 known miRNAs were differentially expressed in this treadmill running model-derived circulating exosome. In the present study, we focused on miR-125a-5p and found that it was indispensable in exercise-induced revascularization *in vitro* and *in vivo*. This conclusion is supported by the following reasons and findings. First, the basic abundance of miR-125a-5p was the highest in circulating exosomes released by exercise or sedentary rats. Second, previous studies have reported that miR-125a-5p could promote the growth, angiogenesis, and metastasis of colorectal and undifferentiated tumors (41, 42). Third, inhibition of miR-125a-5p remarkably decreased the promoting effect of exercise-derived circulating exosomes on EC proliferation and migration. Fourth, 4 weeks of treadmill running-induced collateral growth and angiogenesis were abrogated by using AAV9, which carried a specific sequence targeting miR-125a-5p to inhibit of miR-125a-5p *in vivo*. Inconsistently, one study showed that miR-125a-5p impaired angiogenesis in aging mice (43). We thought that this discrepancy should be due to the different conditions of the ECs used. Moreover, Gao et al., found that miR-125a-5p promotes nasopharyngeal carcinoma growth by targeting TP53 and enhancing the genes associated with cell survival and angiogenesis (41). Conversely, Zhao and Chen et al., showed that miR-125a-5p inhibit angiogenesis by regulating VEGFA or 4EBP-1 in preeclampsia (44) and colorectal cancer (42) respectively. Recently, another paper showed miR-125a-5p impair endotheliogenesis in CD34 + sorted adipose derived stromal/stem cells (45). For these inconsistencies and contradictions phenomena, we imagine that there may be the following reasons: First, the diversity and complexity of biological system and the change of any experimental conditions may lead to the inconsistency of experimental results. Second, it can be said that experimental results are correct and have their own rationality, but we cannot apply simple understanding such as biological model or linear signal pathway to analyze it. Overall, exercise-induced beneficial effects on blood perfusion recovery are mediated, at least in part, by plasma-derived exosomal miR-125a-5p.

ECE1 is a membrane-bound metalloprotease from the M13 zinc-dependent ECE family, which involves three closely related variants: ECE1, ECE2, and ECE3 (46). Their canonical role is to catalyze large ET-1, generating active ET-1, which is ubiquitously synthesized by almost every cell type, with the highest expression in vascular ECs (47). In particular, ET-1 not only exerts a long-lasting and potent vasoconstrictive response by binding to ET_A_ receptors on SMCs, but also causes a decrease proliferation of ECs by binding to ET_B_ receptors (48). Moreover, it has been proposed that ET-1 plays a crucial role in regulating the invasion and angiogenesis of many types of tumors as a mitogen (46). It is intriguing that several clinical studies have shown that plasma ET-1 is implicated in poor collaterals (49) and microvascular dysfunction (50) in CAD patients. However, the biological effects of ET-1 are entirely dependent on its enzymatic activation by ECE1 due to having a very short half-life of approximately 90 s (46). Thus, we speculated that ECE1 may also play a critical role in vascular regeneration. In our current study, we provided solid evidence to support, for the first time, the finding that ECE1 was a functional target of miR-125a-5p promoting angiogenesis and arteriogenesis *in vitro* and *in vivo*. First, 2 putative target sites of miR-125a-5p in the ECE1 mRNA 3′UTR were predicted *via* TargetScan software. Furthermore, we verified that miR-125a-5p could directly bind the 3′UTR of ECE1 by performing a luciferase reporter assay. Second, the protein expression levels of ECE1 were adversely regulated by miR-125a-5p at baseline or with the VEGF stimulation. Likewise, an increased ECE1 protein expression in adductor muscles was closely associated with a reduced miR-125a-5p expression by AAV9-sponge-miR-125a-5p treatment *in vivo*. Functionally, siRNA-ECE1 rescued the inhibitory effects of the miR-125a-5p inhibitor on EC proliferation and migration.

It has been well established that AKT/eNOS phosphorylation and Notch1 activation play paramount roles in key EC functions, such as proliferation, migration, and angiogenesis (51, 52). In the present study, our findings showed that the protein expression of total Notch1, AKT, eNOS and cleaved Notch1 was not changed, whereas the phosphorylation of AKT and eNOS was remarkably increased, in parallel with a reduced ECE1 expression, in ECs incubated with Exe-Exo and VEGF. This suggests that it might be the selective regulation of the VEGF-induced AKT/eNOS signaling pathway and not Notch1 activation are the underlying molecular mechanisms of vascular adaptations in response to exercise training. Furthermore, miR-125a-5p overexpression was associated with a reduced ECE1 expression and an enhanced VEGF-induced AKT/eNOS phosphorylation in an analogous manner as Exe-Exo treatment. In addition, deficiency of miR-125a-5p or ECE1 could counteract the effects of Exe-Exo or miR-125a-5p in the AKT/eNOS signaling pathway, respectively, highlighting the dependency of Exe-Exo and miR-125a-5p on the downstream AKT/eNOS pathway.

Acute exercise can regulate the genes involved in multivesicular body biogenesis, fusion and exosome release, resulting in an increase in the number of exosomes (53). Nevertheless, long-term exercise generally alters the composition of the exosome content (20). We then attempted to decipher the specific tissue source of the circulating exosomal miR-125a-5p. It was determined that miR-125a-5p was abundantly expressed in skeletal muscle and significantly increased in the exercise group, whereas there were no significant differences in other work-related tissues, such as the heart, liver, kidney, aorta and adipose tissue. To some extent, this echoed other reports that skeletal muscle was the main endocrine organ and possessed a distinct secretory property within the circulatory milieu (14, 54). Circulating exosomes packaged with some muscle-specific miRNAs (miR-133b, miR-181a-5p, miR-146a and miR-206) dramatically increased after acute exercise and exerted a versatile role in muscle repair and maintenance (55, 56). Unfortunately, we were unable to define a stimulus that mimics exercise *in vitro* to explore whether skeletal muscle cells could induce miR-125a-5p expression more precisely. As previous studies treated H9C2 cells with IGF-1 (15) or vascular ECs with fluid shear stress (20) to detect cell adaptations to exercise *in vitro*. Overall, based on the present results and previous studies, skeletal muscle was probably the predominant tissue origin for exosomal miR-125a-5p. However, much remains to be elucidated about the cellular or tissue origins and the full complexity of exosome release in response to exercise. Further detailed studies are required in the future.

As discussed above, it remains difficult to identify the precise origin of exercise-derived circulating exosomal miR-125a-5p due to technical limitations. Moreover, the present study cannot exclude the possibility that other circulating exosomal miRNAs with an altered expression, such as miR-23a-3p and miR-365-3p, might confer effects in exercise-induced revascularization, which requires further elucidation.

The past 26 years of research into angiogenic and cell therapies for PAD have not yet translated into significant changes in PAD therapeutics. The reason may be that the animal models used differ greatly from the actual clinical patients. As the animal hindlimb ischemia model is absent of cardiovascular risk factors such as hypertension, diabetes, hypercholesterolemia, senescence or tobacco and alcohol exposure. A more feasible approach may be to preferentially use genetic mouse models that combine cardiovascular risk factors and use modifications to the classic ligation/excision to more closely mimic human conditions (57).

In summary, an important discovery of this work is that comparatively moderate-intensity interval treadmill exercise-derived circulating exosomes synergistically promote arteriogenesis and angiogenesis in ischemic hindlimbs. In addition, we determined that miR-125a-5p is a critical component within Exe-Exo that mediates exercise-induced revascularization. Mechanistically, the molecular bases for these exercise-afforded benefits were partly attributed to the exosomal miR-125a-5p downregulation of ECE1 expression and the subsequent activation of the AKT/eNOS downstream signaling pathway. Moreover, skeletal muscle may be a major tissue source of exercise-induced exosomal miR-125a-5p. These findings present new mechanistic insights into the promoting effect of exercise on revascularization, identify miR-125a-5p as a novel exerkine, and highlight its potential therapeutic role in the prevention and treatment of PAD. Although tested specifically in PAD, arteriogenesis and angiogenesis are common endogenous repair processes observed in a series of ischemic diseases (e.g., CAD, and cerebral infarction), indicating that the therapeutic efficacy of exercise-derived exosomal miR-125a-5p may extend beyond PAD.

## Data availability statement

The datasets presented in this study can be found in online repositories. The names of the repository/repositories and accession number(s) can be found in the article/Supplementary Material.

## Ethics statement

The animal study was reviewed and approved by Animal Care and Use Committee of the Department of Laboratory Animals, Central South University.

## Author contributions

YB, CL, and XQ conceived of the study and designed the experiments. XQ, LL, YXi, ZZ, MC, JZha, BY, and LH performed the experimental work. YB, CL, XQ, JZho, YXi, HY, QS, WW, LZ, and YL analyzed and interpreted the data. XQ wrote the manuscript. YB, CL, JZho, YXu, and LL did critical editing. All authors read and approved of the final version of the manuscript.

## Conflict of interest

The authors declare that the research was conducted in the absence of any commercial or financial relationships that could be construed as a potential conflict of interest.

## Publisher’s note

All claims expressed in this article are solely those of the authors and do not necessarily represent those of their affiliated organizations, or those of the publisher, the editors and the reviewers. Any product that may be evaluated in this article, or claim that may be made by its manufacturer, is not guaranteed or endorsed by the publisher.

## Abbreviations

miRNA: microRNA
Sed-Exo: exosomes purified from plasma of sedentary rats
Exe-Exo: exosomes purified from plasma of exercised rats
PAD: peripheral arterial disease
CAD: coronary artery disease
FAL: femoral artery ligation
GW4869: a well-recognized sphingomyelinase inhibitor to reduce exosome release
DMSO: dimethylsulfoxide
VEGF: vascular endothelial growth factor
ET-1: endothelin-1
ECE1: endothelin converting enzyme 1
AKT: protein kinase B
eNOS: endothelial nitric oxide synthase
UTR: untranslated region
AAV9: serotype 9 adeno-associated virus
NTA: nanoparticle tracking analysis
SMC: smooth muscle cell
EC: endothelial cell
HUVEC: human umbilical vein endothelial cell.
