# Supplementary material for: Prophylactic exercise-derived circulating exosomal miR-125a-5p promotes endogenous revascularization after hindlimb ischemia by targeting endothelin converting enzyme 1
Source: Front Cardiovasc Med. 2022 Jul 22;9:881526. doi: 10.3389/fcvm.2022.881526 (PMC9354753; doi:10.3389/fcvm.2022.881526)
Supplement: Supplementary file 1 [file Data_Sheet_1.docx]

**Supplementary materials and methods**

**Animal protocols**

The animal experiments were divided into five parts. In the first part of the animal study, as shown in Figure S1a, 12 rats were randomly divided into two groups, namely, the Exe and Sed groups. After 4 weeks, all rats were subjected to FAL surgery. Then hindlimb perfusion recovery was measured postoperatively on selected days (0, 3, 7, 14 and 21) via laser Doppler.

In the second part of the animal study (Figure 2a), first, 16 rats were randomly divided into two groups, namely, the Exe and Sed groups. After 4 weeks, all the rats were subjected to FAL surgery and euthanized, and blood was taken from the heart 7 days post-FAL. Second, 1 mg of plasma-derived Sed-Exo or Exe-Exo was mixed with pluronic gel (25% (w/v), Pluronic F127, Sigma) to a final volume of 500 μl and subsequently injected into the adductor muscle as five separate 100-μl injections with a 28-gauge needle. Then, rats were subjected to FAL surgery 3 days after the injection, and the hindlimb perfusion recovery was measured postoperatively on the selected days (0, 7, 14 and 21) via laser Doppler. The rats were sacrificed, and the gastrocnemius and adductor muscles were harvested for determing the expression of miR-125a-5p and the analysis of IHC and IFC 7 days post-FAL.

In the third part of the animal study (Figure 3a), 30 rats were randomly assigned to two groups: Exe+GW4869 and Exe+DMSO. GW4869 (Sigma-Aldrich), dissolved in DMSO, was intraperitoneally (i.p.) injected at one dose of 2 μg/g wt every other day. The same volume of DMSO was injected as controls. Then, all the rats were subjected to 4-weeks of treadmill exercise training followed by FAL surgery, and hindlimb perfusion recovery was measured postoperatively on the selected days (0, 7, 14 and 21) via laser Doppler. To determine whether GW4869 can decrease the concentration of plasma-derived exosomes, 3 rats from each group were euthanized, and blood was taken from the heart 1 week after surgery. Eventually, the rats were sacrificed, and the gastrocnemius and adductor muscles were harvested for the analysis of IHC and IFC 7 days post-FAL.

In the fourth part of the animal study (Figure 5a), AAV represents an efficient and safe vector for in vivo gene transfer, and serotype 9 is significantly cardiotropic and musculotropic. Therefore, we used AAV9-sponge-miR-125a-5p-mCherry to determine whether inhibition of miR-125a-5p impairs exercise-mediated perfusion recovery. Briefly, the rats were randomly assigned to receive adductor injection in situ of either AAV9-sponge-miR-125a-5p or AAV9-control (1*10^12^ vg/ml, 15 μl/point, 5 points) per animal. AAV9 and the control were purchased from Hanbio, Shanghai, China. After 3 weeks, the rats were subjected to in vivo imaging to determine if the virus had entered the tissue for expression. Then, the rats were subjected to 4 weeks of treadmill exercise training followed by FAL surgery and the hindlimb perfusion recovery was measured postoperatively on selected days (0, 7, 14 and 21) via laser Doppler. Eventually, the rats were sacrificed, and the gastrocnemius and adductor muscles were harvested for the analysis of IHC, IFC, western blot and real-time PCR 7 post-FAL.

To determine whether the expression of miR-125a-5p in adductor muscle was influenced by FAL surgery itself, 30 rats were subjected to the FAL surgery. The adductor muscles were harvested on both the ischemic and uninjured sides at selected days (0, 1, 3, 7, 14 and 21) post-surgery to detect the expression of miR-125a-5p via real-time PCR.

**Exosome Isolation from Plasma by Ultracentrifugation**

7 days after the surgery, the rats were euthanized and blood was collected from the left ventricle using a 17-gauge needle and rapidly moved to an EDTA-containing vacutainer (BD Biosciences, USA) to minimize red blood cell damage and platelet activation. Approximately 10-18 ml of blood were obtained from each rat and centrifuged at 1600 xg for 20 min at 4 °C to remove red blood cells and peripheral blood leukocytes. An aliquot (1 ml/tube) of the plasma was immediately frozen and stored at -80 °C for later analysis. The exosomes were isolated from plasma using the ultracentrifugation method^1, 2^. Briefly, plasma was diluted 10 times with 1x PBS and then centrifuged at 10,000 xg for 30 min at 4 °C to remove the cell debris. The supernatant was passed through a 0.22-μm filter (Millipore, MA, USA) and further ultracentrifuged twice at 100,000 xg for 90 min at 4 °C with an SW-41 rotor (Beckman, USA). The exosome pellet was placed at the bottom of the tube and resuspended in 1x PBS (50 µl/1 ml of plasma) for further study.

**Exosome Identification and Labeling**

Exosome identification via transmission electron microscopy (TEM) was performed as previously described^1, 3^. Briefly, freshly isolated exosomes in PBS were transferred to formvar carbon-coated films on copper-grids for 4 min at room temperature (RT), and excess liquid was removed by gently using absorbing paper. After staining with phosphotungstic acid for 1 min and drying for 2 min at RT, the samples were visualized using a Tecnai G2 spirit twin transmission electron microscope (FEI, USA). The size distribution and concentration of the plasma-derived exosomes were analyzed by NanoSight running nanoparticle tracking analysis (NTA) software (ZataView). The presence of the exosomal marker proteins TSG101 and CD63 and the negative expression of calnexin were measured by western blot. Purified exosomes were labeled with the green fluorescent dye PKH67 (Sigma), as previously described^4^. Exosomes from 2 ml plasma were resuspended in 0.5 mL of Diluent C, followed by 4 μl of PKH67. After 4 min of incubation at RT in the dark, 2 mL of exosome-free fetal bovine serum (FBS) were added to terminate the labeling reaction, and then washed twice with PBS by centrifugation (100,000 xg, 1 hour, 4 °C) to remove the remaining dye. The exosomes were suspended in a basal medium and incubated with HUVECs for 24 h at 37 °C. The cells were washed three times with PBS, fixed with 4% paraformaldehyde in PBS for 20 minutes at RT and then stained with phalloidin (Abcam, ab176757) for 45 min and 0.1 g/mL DAPI (4-6-diamidino-2-phenylindole) for 10 min. The cells were analyzed using a fluorescence microscope (Leica). For the localization of exosome after injection in vivo, 1 mg of Sed-Exo or Exe-Exo mixed with pluronic gel was injected into the adductor muscle by five separate injections with a 28-gauge needle. After 24 h, exosome signal was detected by immunofluorescence in adductor muscle.

**Laser Doppler based hindlimb perfusion measurement**

Blood flow to the ischemic or nonischemic hindlimb was assessed using a Pericam laser Doppler system (PeriCam PSI Z, Perimed, Sweden) as previously described^5, 6^. The animals were anesthetized and their body temperatures were strictly maintained between 36.5 °C and 37.5 °C. The hindlimb blood flow was measured postoperatively on selected days (0, 3, 7, 14 and 21). The level of perfusion in the ischemic and unoperated hindlimbs was quantified using the mean pixel value within the region of interest, and the relative changes in the hindlimb perfusion recovery were expressed as the ratio of the ischemic over unoperated laser Doppler-detected blood perfusion.

**Agomir-****125a-5p transfection in vivo**

The transfection of agomir-125a-5p (2'OME+5'chol modified) (Ribobio, China) and agomir-NC was performed as previously described^5^ . Briefly, agomiR oligonucleotide was synthesized in vivo (Ribobio, China), then deprotected, desalted, and purified by high-performance liquid chromatography. Rats received agomirs (10 nmol) by multi-point injections in the left gastrocnemius immediately after femoral artery ligation. The injections were repeated everyday till 7 days after the operation. The gastrocnemius were collected on day 7 for further experiments for angiogenesis.

**Immunohistochemistry (IHC) and immunofluorescence (IFC)**

A histological analysis was performed on perfusion/fixed skeletal muscles (adductor and gastrocnemius) collected from rats on the 7th day after femoral artery ligation. The formalin‑fixed tissues were embedded in paraffin wax and cut into 4-µm sections. The tissue was stained with hematoxylin and eosin (H&E) for a histological analysis. Immunohistochemistry was then used to stain the vessels. Collateral arteries were labeled with an α-SMA antibody (1:400, Abcam, ab5694), and capillaries were stained using a CD31 antibody (1:100, Abcam, ab9498). Images were taken via microscope at 40× magnification. For the measurement of proliferating arterial smooth muscle and endothelial cells, we performed immunofluorescence double staining for Ki67 (1:100, Abcam, ab1667) and CD31 (1:50, Abcam, ab9498), Ki67 (1:100, Abcam, ab1667) and α-SMA (1:100, Abcam, ab5694), respectively. Secondary antibodies were replaced with antibodies labeled with Alexa Fluor (1:200, Abcam, England) dye with a maximum excitation at 488 nm (green), and for red with Alexa Fluor 594 nm. The slides were counterstained with DAPI to visualize the cellular nuclei. The immunofluorescence slices were scanned using a Pannoramic Scanner (3D HISTECH, Hungary) with CaseViewer 2.0 scanning software, and pictures were taken at 40x and 90x magnifications and analyzed by imageJ.

**Exosomal miRNA library preparation and sequencing**

Exosomal miRNA sequencing and analysis were conducted using a commercial service (OE Biotechnology, Shanghai, China). Briefly, the total RNA was extracted from exosomes purified via ultracentrifugation from 3 ml of plasma and quantified using a NanoDrop ND-2000 (Thermo Fisher Scientific). Both 3' and 5' adaptors were added to each end, followed by reverse transcription and PCR amplification. The PCR products were purified via electrophoresis and sequenced using the Illumina HiSeq 2500 platform. Differential expression of the miRNAs between the 2 groups was analyzed using a cluster analysis. The miRNAs with a level change of greater than 2-fold (up- or downregulated compared with the sedentary group) (p < 0.05) were screened out, and RT-qPCR was performed to validate the expression changes of miRNAs^1, 7^.

**RNA Isolation and Quantitative Real Time-qPCR (RT-qPCR)**

The total RNA was extracted from exosome pellets, cells and adductor muscle using a TRIzol-based (Invitrogen, USA) RNA isolation protocol, as previously described^8^. To improve the efficiency of exosomal RNA extraction, a Dr. GenTLE Precipitation Carrier (TaKaRa, Japan) was added following the isopropanol step. Real-time PCR was performed by using SYBR Premix Ex Taq RT-qPCR assays in an ABI 7500 real-time PCR system (Life Technologies, USA). For the detection of cells and tissues, U6 was used as an internal standard. The primer sequences for U6 were as follows: 5′-CTCGCTTCGGCA GCACA-3′ (forward) and 5′-AACGCTTCACGAATTTGCGT-3′ (reverse). For exosomal miRNA detection, the synthetic miRNA Caenorhabditis elegans miR-39 (cel-miR-39; 5 fmol/μl; 1μl/sample; sequence: 5ʹ-UCACCGGGUGUAAAUCAGCUUG-3′; Ribobio, China) was added to the isolated RNAs and was used as an exogenous control. The relative expression level of miRNA was normalized to U6 or cel-miR-39 and shown as a ratio relative to the indicated controls using the 2-ΔΔCt method.

**Nano-flow cytometry analysis of exosomes**

In order to detect the exosome subspecies, we performed nano-flow cytometry by using the specific surface proteins of the exosomes. Briefly, exosomes were isolated from 3 ml of plasma using the ultracentrifugation method and incubated with CD31-BB700 (BD Pharmingen, #746024), CD61-FITC (BD Pharmingen, #561909) and the corresponding isotype and negative controls for 30 min at 37 °C in the dark, according to the manufacturer’s protocols. Each sample was divided into seven groups for staining: CD31, CD61, CD31+CD61, FITC, BB700, CD31+FITC and CD61+BB700. To determine the probable origin of plasma-derived exosomes, a Flow NanoAnalyzer (NanoFCM) was used. The sorting was gated for vesicles between 30–150 nm in diameter. CD31^+^/CD61^−^, CD31^+^/CD61^+^, CD31^−^/CD61^−^ and CD31^−^ /CD61^+^ exosomes were gated as previously describe^7^. The percentages of each group were recorded for the analysis.

**Cell culture**

Human umbilical vein endothelial cells (HUVECs) were purchased from ScienCell Research Laboratories and cultured on an endothelial cell medium (ECM, ScienCell, Cat. No. 1001) supplemented with 10% fetal bovine serum (FBS, Cat. No. 0025), 1% endothelial cell growth supplement (ECGS, Cat. No. 1052) and 1% antibiotic solution (P/S, Cat. No. 0503) in a 37 °C, 5% CO_2_ incubator. For cell treatment, 50 μg/ml exosomes were added to the medium. Either miR-125a-5p mimic (50 nM), mimic-NC (50 nM), miR-125a-5p inhibitor (100 nM), inhibitor-NC (100 nM) or ECE1 siRNA was transfected into the HUVECs for 48 hours using Lipofectamine 2000 (Invitrogen, USA) according to the manufacturer's instructions. The cells were then starved for 6 hours, followed by stimulation with VEGF165 (100 ng/ml, Peprotech, USA) for 20 minutes.

**Western blotting**

The proteins were exracted from cells and exosome pellets using a RIPA lysis buffer containing protease and phosphatase inhibitors (Sigma). The protein concentration was measured using a BCA assay (Beyotime, Beijing, China) and used for loading of 20 to 60 μg on 10% SDS/PAGE gels. Then the gels were subjected to electrophoresis and transferred to a polyvinylidene difluoride membrane. The membranes were blocked with 5% BSA or non-fat milk for 1 hour and incubated with the primary antibodies overnight at 4 °C, followed by incubation with the corresponding secondary antibodies. Antibodies against phosphor(p)-eNOS (Ser1177) (AF3247, 1:1000), eNOS (AF0096, 1:1000), p-AKT(Ser473) (AF0016, 1:1000) and AKT (AF6261, 1:1000) were purchased from Affinity Bioscience (Melbourne, Australia). Antibodies against Notch1 (#3608, 1:1000) and cleaved-Notch1 (#4147, 1:1000) were purchased from Cell Signaling Technology (Danvers, USA). Antibodies against GAPDH (ab181602, 1:5000), TSG101 (ab125011, 1:5000) and calnexin (ab22595, 1:250) were purchased from Abcam (Cambridge, USA). Antibodies against ECE1 (sc-376017, 1:500) and CD63 (sc-5275, 1:1000) were purchased from Santa Cruz Biotechnology (California, USA). The bands were visualized using prosignal pico ECL reagent (Prometheus Protein Biology Products) in a ChemiDoc™ Imager (Bio-Rad). GAPDH was used as an internal reference. The relative intensity of the immunoreactive bands was determined via IntDen using ImageJ software.

**Measurement of EC Proliferation, Migration and Tube Formation**

HUVECs proliferation was determined by CCK-8. After transfection or exosome incubation, the cells were seeded (5 X 10^3^ cells/well) in 96-well plates and incubated at 37 °C and 5% CO2 overnight. Then, 10 μl of the CCK­8 solution was added to each well and incubated for 2 hours. The absorbance of the plate was measured at 450 nm with a microplate reader (Molecular Devices, Menlo Park, CA). The migration of HUVECs was determined by performing a cell scratch assay. First, on the back of the 6-well plate, three horizontal lines were drawn with a marker pen. Then, the HUVECs were seeded on 6-well plates, and cell treatment was performed when the confluence reached approximately 70%. Three vertical lines were drawn in the plate with a 10 μl sterile tip, and the suspended cells were washed with PBS. After 24 hours of culture, the cells were photographed at the field where the two vertical and horizontal lines intersect under an inverted microscope. the formation of vessel-like structures by HUVECs on growth factor-reduced Matrigel (BD Biosciences) was performed as previously described^9^. And the degree of tube formation was quantified by measuring the total tube length per fields (X 20) from each dish using ImageJ.

**Luciferase Reporter Assay**

A luciferase reporter assay was performed as previously described^10^. A coding-region fragment containing the miR-125a-5p binding site was generated via PCR and cloned into the pmiR-RB-REPORT^TM^ luciferase vector (Ambion). Two mutations in the binding site seed sequence in the ECE1 mRNA 3′-UTR were introduced. A DNA fragment containing the 3′-UTR miR-125a-5p binding site or mutation site was sequenced to confirm the identity. Then, by using Lipofectamine 2000 (Invitrogen), plasmid constructs (WT-3′-UTR, MT1-3′-UTR and MT2-3′-UTR) (250 ng/well) were co-transfected into 293T cells with miR-125a-5p mimic (50 nM) and a negative control (50 nM) in 96-well plates. After transfection for 48 h, luciferase assay reagents (Dual-­Glo®Luciferase Assay System, Promega, USA) were added to measure luciferase activity by an illuminometer (Veritas 9100-002).

**table S1. Differentially expressed miRNAs**

| miRNA_ID Sed-Exo Exe-Exo Fold change *p* value up/down sequence |
| --- |
| rno-miR-125a-5p 1630.59 4329.86 2.66 0.026007 up 5'-TCCCTGAGACCCTTTAACCTGTGA-3'  rno-miR-128-3p 0.69 161.53 233.48 0.005692 up 5'-TCACAGTGAACCGGTCTCTTT-3'  rno-miR-206-3p 0 129.46 infinite 0.001973 up 5'-TGGAATGTAAGGAAGTGTGTGG-3'  rno-miR-23a-3p 299.41 864.24 2.89 0.04267 up 5'-ATCACATTGCCAGGGATTTCC-3'  rno-miR-362-5p 1.13 121.47 107.26 0.02688 up 5'-AATCCTTGGAACCTAGGTGTGAAT-3'  rno-miR-365-3p 0 82.21 infinite 0.033385 up 5'-TAATGCCCCTAAAAATCCTTAT-3'  novel169_mature 0 98.51 infinite 0.011336 up 5'-TGAAAGGGAAGCGCTTGTG-3'  novel333_mature 0 78.72 infinite 0.043552 up 5'-ACGGGAGGGGCAGAGGGA-3'  rno-miR-652-3p 92.56 1.06 0.01 0.048159 down 5'-AATGGCGCCACTAGGGTTGTG-3'  novel25_mature 994.43 270.03 0.27 0.013881 down 5'-TGAGGCAGCAGTGGGGAA-3'  novel26_mature 129.35 2.81 0.02 0.023407 down 5'-TGTGGCTCAAATGGTAGA-3'  novel54_mature 339.62 0 0 0.006962 down 5'-ACCTTGGTGGAACTGTGTC-3'  novel69_mature 155.67 0.26 0.002 0.000574 down 5'-CCTGGCCAGGCGGTGGTGACC-3'  novel72_mature 298.37 33.41 0.11 0.007897 down 5'-GGGGAAGAGCCCAACTTGAA-3'  novel157_mature 91.29 0 0 0.014768 down 5'-ACAGCACTGTAGAGGCAGG-3'  novel163_mature 110.12 0 0 0.004849 down 5'-AATTCTGGTTTCTGGCAC-3'  novel189_mature 192.44 2.63 0.01 0.001909 down 5'-GCTGAGAGACGAGAGCTAC-3'  novel218_mature 113.36 0.21 0.002 0.006958 down 5'-TGCTGTAGCTCAGTGGTAGAGC-3'  novel225_mature 210.20 25.20 0.12 0.030681 down 5'-GGGCTTGGAAGAATCAGT-3'  novel316_mature 145.56 0.26 0.002 0.004681 down 5'-CCATGGTCAGGATGAAGCT-3' |

Exe-Exo: exosomes isolated from plasma of exercised rats. Sed-Exo: exosomes isolated from plasma of sedentary rats. Fold change indicates Exe-Exo vs. Sed-Exo.

**Supplementary Figures**

**
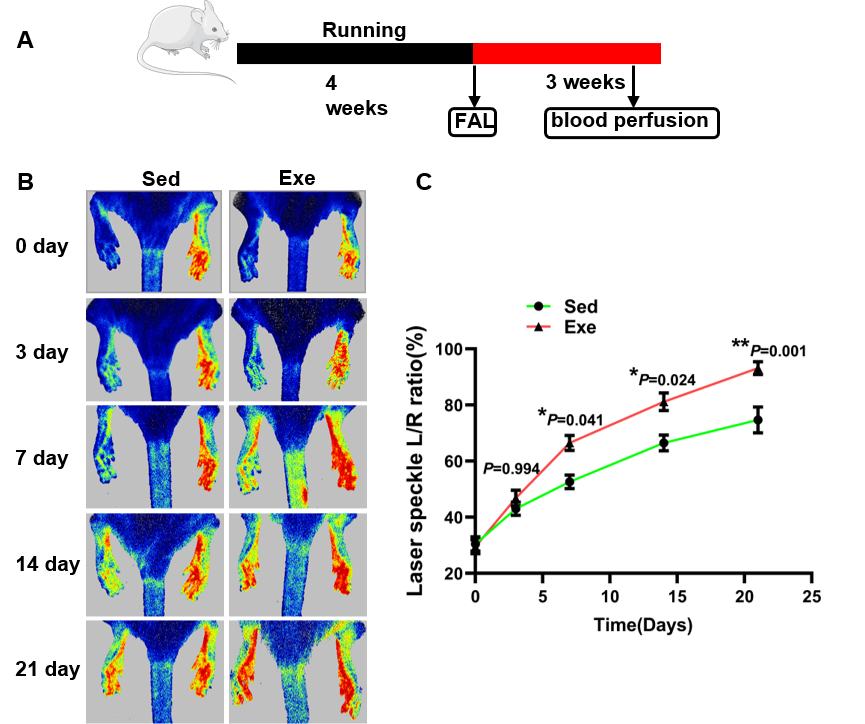
**

**Figure S1. Prophylactic exercise increases hindlimb perfusion recovery. (A).** Time course of the experimental setup. **(B and C).** Representative laser speckle perfusion images **(B)** and quantitative analysis **(C)** of the ratios of left to right (L/R) hindlimb blood perfusion between Sed- and Exe-exposed rats at the indicated times after FAL. N = 6 per group. **P*<0.05, ***P*<0.01, Sed vs. Exe. Sed, sedentary. Exe, exercise. FAL indicates femoral artery ligation. Data are means ± SEM.


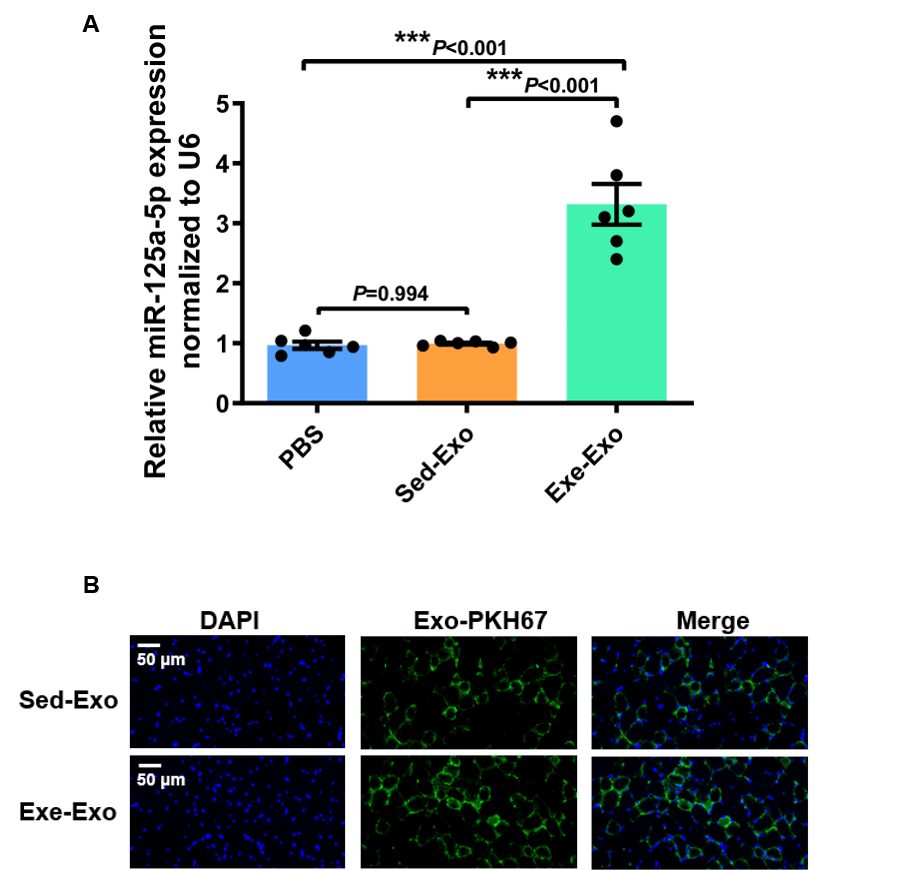


**Figure S2.** **The internalization of plasma exosomes by adductor muscle.** Equivalent quantities of exosomes (1 mg, mixed with pluronic gel) isolated from the plasma of rats (Exe or Sed) 7 days post-FAL were injected into the left adductor muscles of the second batch of healthy adult rats. **(A).** The second batch rats were exposed to FAL and adductor muscles were obtained 7 days after the surgery. The expression of miR-125a-5p in adductor muscle was measured by RT-PCR. **(B).** Exosome signal was detected by immunofluorescence in adductor muscle 24 hours after the injection. Green, PKH67-labeled exosomes. FAL indicates femoral artery ligation. N = 6 per group. ****P*<0.001. Data are means ± SEM.


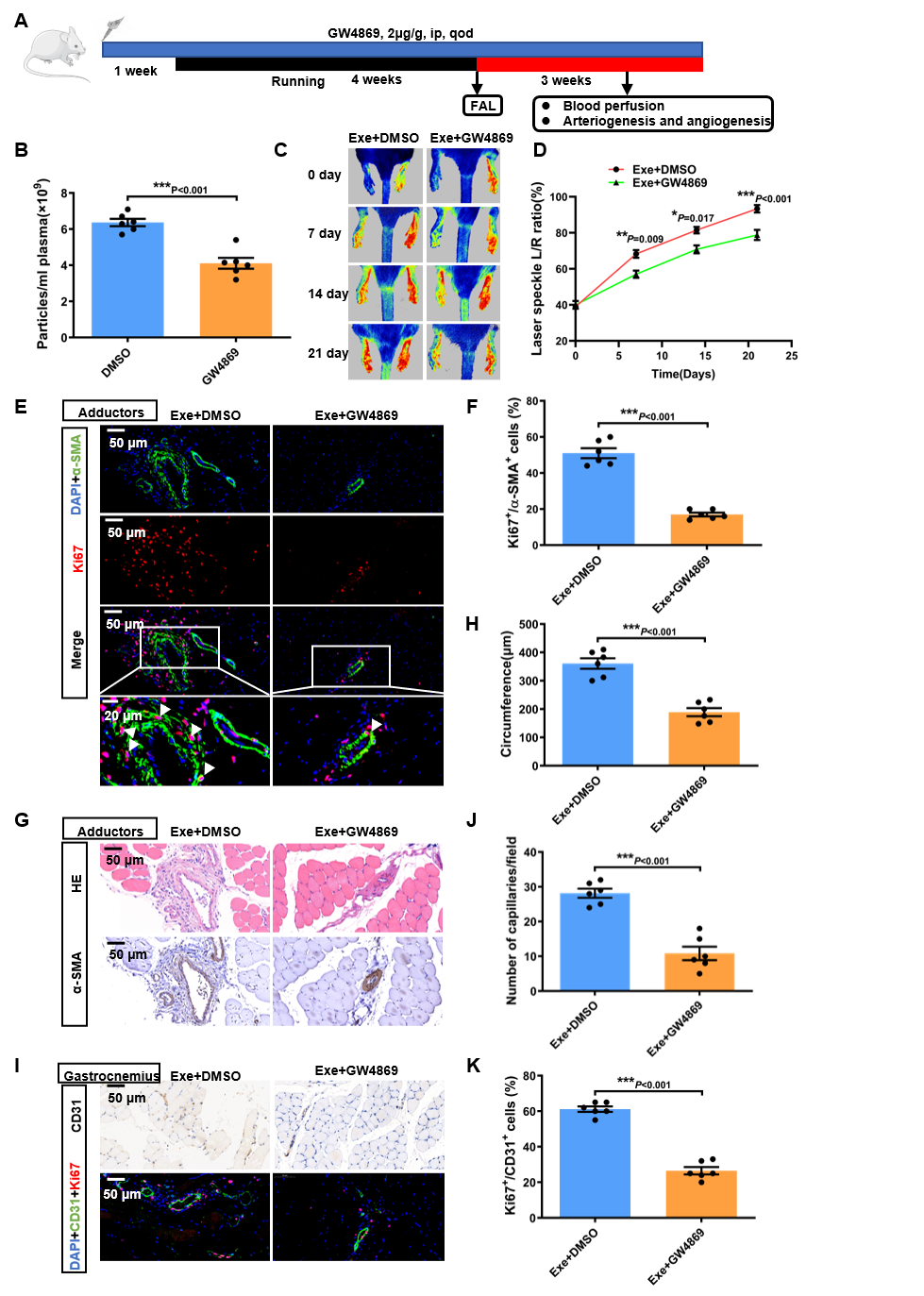


**Figure S3. Inhibition of exosome release with GW4869 counteracts pro-arteriogenesis and angiogenesis induced by exercise. (A).** Rats were pretreated with an exosome-release inhibitor (GW4869, 2 μg/g wt, intraperitoneal and once every other day) 1 week before the 4-week exercise training and then subjected to FAL surgery. **(B).** Quantitative analysis of the effect of GW4869 on the concentration of plasma-derived exosomes. **(C and D).** Representative laser speckle perfusion images **(C)** and quantitative analysis **(D)** of the ratios of left to right (L/R) hindlimb blood perfusion among Exe+DMSO- and Exe+GW4869- treated rats at the indicated times after FAL. **(E and F).** Representative images of immunofluorescence double staining **(E)** and a quantitative analysis **(F)** of the cross-sections of adductor muscles 7 days after the surgery. Red, Ki67. Green, α-SMA. Blue, DAPI. **(G and H).** Representative images **(G)** and quantitative analysis **(H)** of HE staining and immunohistochemistry of α-SMA in the cross-sections of the adductor muscles from Exe+DMSO- and Exe+GW4869- treated rats 7 days after surgery. **(I-K).** Representative images **(I)** and quantitative analysis **(J and K)** of immunofluorescence double staining and CD31 immunohistochemistry in the cross-sections of the gastrocnemius muscles from Exe+DMSO- and Exe+GW4869- treated rats 7 days after the surgery. The ratio of Ki67-positive cells to the total ECs in each field was quantified**.** Red, Ki67. Green, CD31. Blue, DAPI. Arrows, representative Ki67 positive SMCs. N = 6 per group. **P*<0.05, ***P*<0.01, ****P*<0.001. Exe+DMSO vs. Exe+GW4869. Exe, exercise. FAL indicates femoral artery ligation. Scale bar = 20 or 50 μm, as presented in the above images. Data are means ± SEM.

**
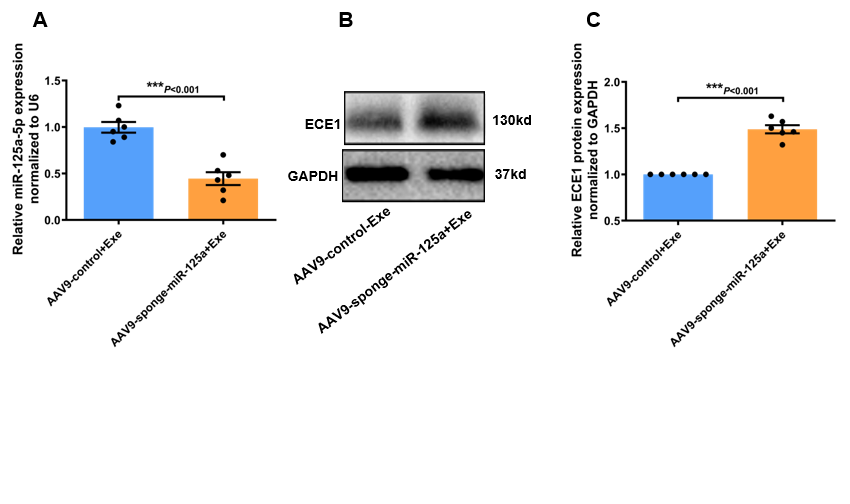
**

**Figure S4.** **Inhibition of miR-125a-5p via AAV9 increased ECE1 protein expression in vivo.** Adductor muscles were collected from rats 7 days post-FAL following AAV9 treatment and exercise training. **(A).** Quantitative analysis of the effect of AAV9 on the expression of miR-125a-5p by RT-PCR. N = 6 per group. Representative images **(B)** and quantitative analysis **(C)** of ECE1 expression determined by western blotting. Western blotting was repeated 6 times. ****P*<0.001. Data are means ± SEM.

**
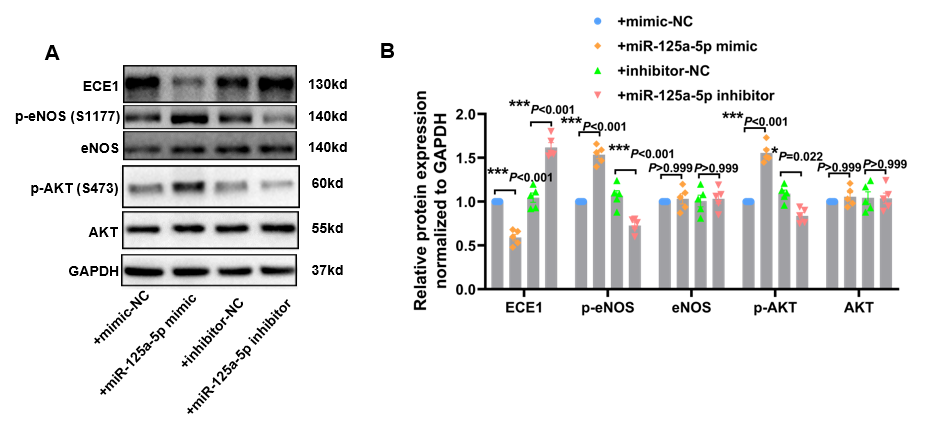
**

**Figure S5. MiR-125a-5p activating AKT/eNOS signaling pathway in HUVECs.** Cells were transfected with miR-125a-5p mimic (50 nM) or inhibitor (100 nM) and the corresponding negative control (NC) for 48 hours, starvated for 6 hours and stimulated with VEGF165 (100 ng/ml) for 20 minutes. Overexpression of miR-125a-5p downregulated ECE1 expression and activated AKT and eNOS phosphorylation. Representative blots **(A)** and a quantitative analysis **(B)** of ECE1, AKT, p-AKT, eNOS and p-eNOS protein expression determined by western blotting. *P<0.05, ***P<0.001. Western blot experiment was repeated 5 times. Data are means ± SEM.

**
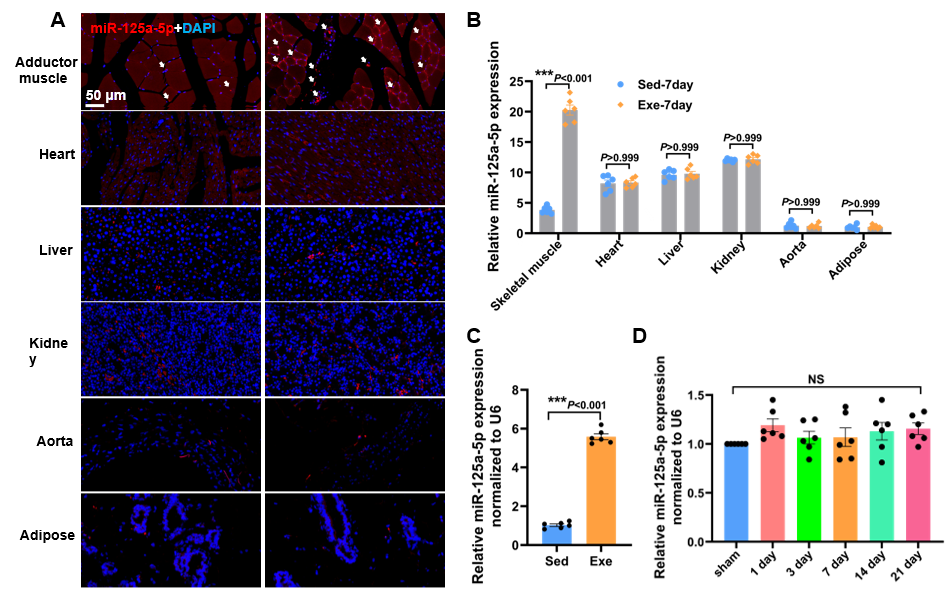
**

**Figure S6. Exercise increases the expression of miR-125a-5p in skeletal muscles. (A+B).** FISH analysis and quantification of miR-125a-5p in adductor muscle, heart, liver, kidney, aorta and adipose tissue collected from rats 7 days post-FAL following 4 weeks of running. Scale bar = 50 μm. Arrows, representative FISH imaging points of miR-125a-5p. Statistical comparisons: Student's *t* test. **(C).** The expression of miR-125a-5p in adductor muscle 24 hours after 4 weeks of exercise training by RT-PCR. Statistical comparisons: Student's *t* test. **(D).** The expression of miR-125a-5p in adductor muscle at the indicated times after FAL by RT-PCR. Statistical comparisons: One-way ANOVA with Tukey’s *post- hoc* analysis. FAL indicates femoral artery ligation. Sed, sedentary. Exe, exercise. N = 6 per group. ***P<0.001. NS indicates no significance for sham vs. multi-time points and Sed-7day vs. Exe-7day. Data are means ± SEM.

**
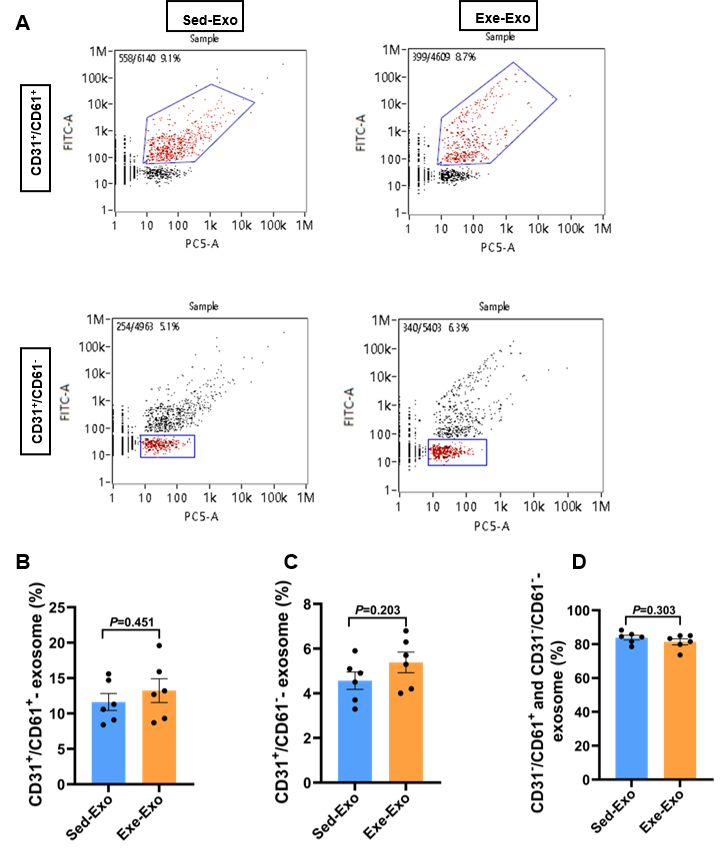
**

**Figure S7. Nanoflow cytometry analysis of Sed-Exo and Exe-Exo showing no significant difference in the origin of endothelial cells and platelets.** **(A).** Representative results of nanoflow cytometry analysis of Sed-Exo and Exe-Exo. **(B-D).** Quantification of the ratio of plasma-derived exosomes from endothelial cells (CD31^+^/CD61^−^), platelets (CD31^+^/CD61^+^) and other types of cells (CD31^−^/CD61^−^ and CD31^−^ /CD61^+^). N = 6 per group. The data are means ± SEM.

**
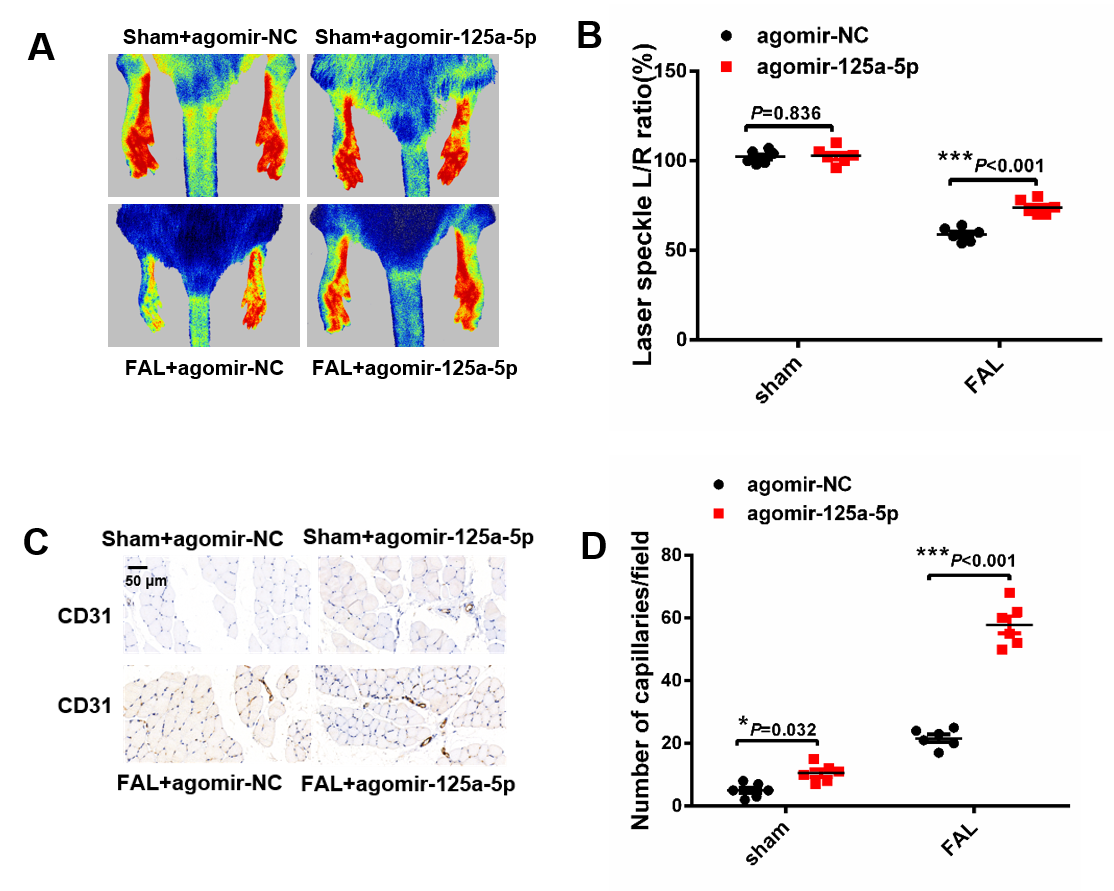
**

**Figure S8. Ischemic and healthy lower limbs responses to miR-125a-5p in vivo.**

**(A and B).** Representative laser speckle perfusion images (A) and a quantitative analysis (B) of the ratios of left to right (L/R) hindlimb perfusion among the rats of sham and 7 days after FAL. **(C and D).** Representative images (C) and quantitative analysis (D) of CD31 immunohistochemistry in cross-sections of the gastrocnemius muscles among the rats of sham and 7 days after FAL. N = 6 per group. FAL indicates femoral artery ligation. Scale bar = 50 μm, as presented in the above images. Data are means ± SEM.

**Reference**

1. Hou Z, Qin X, Hu Y, Zhang X, Li G, Wu J, et al. Longterm Exercise-Derived Exosomal miR-342-5p: A Novel Exerkine for Cardioprotection. *Circ Res*. (2019) 124:1386-1400. doi: 10.1161/CIRCRESAHA.118.314635
2. Gunasekaran M, Sharma M, Hachem R, Bremner R, Smith MA, Mohanakumar T. Circulating Exosomes with Distinct Properties during Chronic Lung Allograft Rejection. *J Immunol*. (2018) 200:2535-2541. doi: 10.4049/jimmunol.1701587
3. Lasser C, Eldh M, Lotvall J. Isolation and characterization of RNA-containing exosomes. *J Vis Exp.* (2012) 9:e3037. doi: 10.3791/3037
4. van Balkom BW, de Jong OG, Smits M, Brummelman J, den Ouden K, de Bree PM, et al. Endothelial cells require miR-214 to secrete exosomes that suppress senescence and induce angiogenesis in human and mouse endothelial cells. *Blood.* (2013) 121:3997-4006. doi: 10.1182/blood-2013-02-478925
5. Zhu LP, Zhou JP, Zhang JX, Wang JY, Wang ZY, Pan M, et al. MiR-15b-5p Regulates Collateral Artery Formation by Targeting AKT3 (Protein Kinase B-3). *Arterioscler Thromb Vasc Biol.* (2017) 37:957-968. doi: 10.1161/ATVBAHA.116.308905
6. Meng S, Gu Q, Yang X, Lv J, Owusu I, Matrone G, et al. TBX20 Regulates Angiogenesis Through the Prokineticin 2-Prokineticin Receptor 1 Pathway. *Circulation.* (2018) 138:913-928. doi: 10.1161/CIRCULATIONAHA.118.033939
7. Ge X, Guo M, Hu T, Li W, Huang S, Yin Z, et al. Increased Microglial Exosomal miR-124-3p Alleviates Neurodegeneration and Improves Cognitive Outcome after rmTBI. *Mol Ther.* (2020) 28:503-522. doi: 10.1016/j.ymthe.2019.11.017
8. Zeng Y, Yao X, Liu X, He X, Li L, Liu X, et al. Anti-angiogenesis triggers exosomes release from endothelial cells to promote tumor vasculogenesis. *J Extracell Vesicles.* (2019) 8:1629865. doi: 10.1080/20013078.2019.1629865
9. Li CC, Qiu XT, Sun Q, Zhou JP, Yang HJ, Wu WZ, et al. Endogenous reduction of miR-185 accelerates cardiac function recovery in mice following myocardial infarction via targeting of cathepsin K. *J Cell Mol Med.* (2019) 23:1164-1173. doi: 10.1111/jcmm.14016
10. Bai YP, Zhang JX, Sun Q, Zhou JP, Luo JM, He LF, et al. Induction of microRNA-199 by Nitric Oxide in Endothelial Cells Is Required for Nitrovasodilator Resistance via Targeting of Prostaglandin I2 Synthase. *Circulation*. (2018) 138:397-411. doi: 10.1161/CIRCULATIONAHA.117.029206
